# Spatial resolution enhancement using deep learning improves chest disease diagnosis based on thick slice CT

**DOI:** 10.1038/s41746-024-01338-8

**Published:** 2024-11-23

**Authors:** Pengxin Yu, Haoyue Zhang, Dawei Wang, Rongguo Zhang, Mei Deng, Haoyu Yang, Lijun Wu, Xiaoxu Liu, Andrea S. Oh, Fereidoun G. Abtin, Ashley E. Prosper, Kathleen Ruchalski, Nana Wang, Huairong Zhang, Ye Li, Xinna Lv, Min Liu, Shaohong Zhao, Dasheng Li, John M. Hoffman, Denise R. Aberle, Chaoyang Liang, Shouliang Qi, Corey Arnold

**Affiliations:** 1 College of Medicine and Biological Information Engineering, Northeastern University, Shenyang, Liaoning China; 2 Key Laboratory of Intelligent Computing in Medical Image, Ministry of Education, Northeastern University, Shenyang, Liaoning China; 3 Infervision Medical Technology Co., Ltd, Beijing, China; 4 National cancer institute, National Institutes of Health, Bethesda, MD USA; 5 Department of Radiological Sciences, University of California Los Angeles David Geffen School of Medicine, Los Angeles, CA USA; 6 Academy for Multidisciplinary Studies, Beijing National Center for Applied Mathematics, Capital Normal University, Beijing, China; 7 Chinese Academy of Medical Sciences & Peking Union Medical College, Beijing, China; 8 Peking University China-Japan Friendship School of Clinical Medicine, Beijing, China; 9 Department of Radiology, Chinese PLA General Hospital First Medical Center, Beijing, China; 10 Department of Radiology, Beijing Haidian Section of Peking University Third Hospital, Beijing, China; 11 Department of Radiology, General Hospital of Ningxia Medical University, Yinchuan, Ningxia China; 12 Department of Radiology, Beijing Chest Hospital, Capital Medical University, Beijing, China; 13 Department of Radiology, China-Japan Friendship Hospital, Beijing, China; 14 Department of Thoracic Surgery, China-Japan Friendship Hospital, Beijing, China; 15 Department of Radiological Sciences, Pathology & Laboratory Medicine, Electrical & Computer Engineering, and Bioengineering, University of California Los Angeles, Los Angeles, CA USA

**Keywords:** Radiography, Three-dimensional imaging, Diagnosis

## Abstract

CT is crucial for diagnosing chest diseases, with image quality affected by spatial resolution. Thick-slice CT remains prevalent in practice due to cost considerations, yet its coarse spatial resolution may hinder accurate diagnoses. Our multicenter study develops a deep learning synthetic model with Convolutional-Transformer hybrid encoder-decoder architecture for generating thin-slice CT from thick-slice CT on a single center (1576 participants) and access the synthetic CT on three cross-regional centers (1228 participants). The qualitative image quality of synthetic and real thin-slice CT is comparable (*p* = 0.16). Four radiologists’ accuracy in diagnosing community-acquired pneumonia using synthetic thin-slice CT surpasses thick-slice CT (*p* < 0.05), and matches real thin-slice CT (*p* > 0.99). For lung nodule detection, sensitivity with thin-slice CT outperforms thick-slice CT (*p* < 0.001) and comparable to real thin-slice CT (*p* > 0.05). These findings indicate the potential of our model to generate high-quality synthetic thin-slice CT as a practical alternative when real thin-slice CT is preferred but unavailable.

## Introduction

Slice thickness of computed tomography (CT) constitutes a vital determinant of image quality, which controls the spatial resolution of the volumetric image. Thinner slices yield images with higher spatial resolution, facilitating the detection of abnormalities, the evaluation of intricate anatomical structures, and the characterization of lesions^1–3^. For instance, in scenarios involving incidental pulmonary nodules, recent guidelines recommend reconstructing chest CT with contiguous thin-slice (thickness ≤ 1.5-mm, typically 1-mm) to enable precise characterization and measurement of small nodules^4^. Despite the diagnostic superiority of high-resolution thin-slice CT, their broad clinical adoption is hampered by the substantial financial burden of acquiring high-quality CT scanners and establishing the necessary data storage infrastructure. Notably, many CT scanners can acquire thin slices; however, reconstruction and storage protocols often default to thick-slice settings, adjustments to which are not straightforward and scanner-specific. This predicament is particularly pronounced in real-world clinical settings of many developing countries^5,6^, where transitioning to advanced CT scanners and establishing large-scale data centers is a complex and resource-intensive endeavor. Consequently, thick-slice CT, typically with a slice thickness of 5-mm, remain the prevalent choice in such regions. The coarse spatial resolution of these thick-slice CT may obscure subtle anatomical features, increasing the likelihood of misdiagnosis or unforeseen consequences^7^.

Another realm susceptible to the influence of slice thickness is computer-aided medical image analysis. Deep learning (DL), an artificial intelligence (AI) subfield, has emerged as the dominant technology in computer-aided medical image analysis, with broad applications in various tasks such as disease diagnosis, lesion detection, and region of interest segmentation^8^. Currently, numerous DL-based algorithms have advanced from prototypes to commercially available products, having successfully undergone stringent rigorous approvals by authoritative bodies such as the United States Food and Drug Administration (FDA) and China National Medical Products Administration (NMPA)^9,10^. These regulated AI-assisted diagnosis products hold immense potential for integration into clinical practice. However, several AI products are developed around thin-slice high-quality images and exhibit suboptimal performance when applied to thick-slice images^11–14^. The aforementioned developing countries, in particular, face significant disparities in accessing and benefiting from AI products, exacerbating the existing healthcare inequalities. Therefore, it is promising to develop a method to translate thick-slice CT into synthetic thin-slice CT with higher spatial resolution, thus narrow the application gap with thin-slice CT.

The advancement of DL promotes its broadly adoption for medical image translation^15–20^. Several studies have demonstrated the feasibility of using DL to use super-resolution (SR) algorithms to enhance spatial resolution of thick-slice CT, generating synthetic thin-slice counterparts —— a process known as “spatial SR”. Early methods were inspired by natural image SR and primarily developed models using convolutional neural networks (CNN) architectures^21–23^. Recently, Yu et al.^24^ proposed a Transformer-based spatial SR method to overcome the inherent shortcomings of the CNN model and obtain state-of-the-art (SOTA) quantitative performance. Although the image quality of DL-based synthetic thin-slice CT shows an increasing trend, the absence of comprehensive multicenter validation poses a barrier to the clinical application of such synthetic medical images.

The purpose of this cross-regional multicenter study was to develop a deep learning synthetic (DLS) model for generating synthetic thin-slice (1 mm) CT from thick-slice (5 mm) CT, and assess the potential of integrating these synthetic thin-slice CT into clinical workflow. The synthetic thin-slice CT was evaluated regarding quantitative metrics, qualitative multi-reader assessment, and diagnostic application for chest diseases. Additionally, we explored can synthetic thin-slice CT improve the performance of regulated AI-assisted diagnosis products that previously underperformed on original thick-slice CT.

We organize the rest of paper to include the following: We first present the demographics of participants and the workflow of our DLS model, then provide evaluation results of synthetic thin-slice CT and assess its performance in diagnosing chest diseases when used by radiologists or AI-assisted products (Results). In the Discussion, we point the challenges of using thick-slice CT for diagnosis, explore how our DLS model enhances the diagnostic capability of thick-slice CT for chest diseases, and discuss the study’s limitations and contributions. Finally, we review related work on spatial SR, detail the architecture of our DLS model, describe the processes for assessing image quality and clinical applicability, and outline the evaluation metrics and statistical analysis (Methods).

## Results

### Dataset characteristics

This multicenter, retrospective study included 2802 participants from four cross-regional centers between April 2015 and July 2022. The study population characteristics are summarized in Table 1. Dataset-Development (Beijing Haidian Hospital, China) included 1576 participants (683 female [43.3%]; median [interquartile ranges (IQRs)] age, 26 [22–33]), of which 1000 (63.5%) were used for training, 176 (11.2%) for validation, and 400 (25.4%) for internal testing. Dataset-USA (University of California Los Angeles Hospital, USA) was a physical examination cohort of older adults, consisting of 174 participants (83 female [47.7%]; median [IQRs] age, 63 [54–71]) who may be healthy or may have various abnormalities. Dataset-Pneumonia (Chinese PLA General Hospital First Medical Center, China) included 300 participants (91 female [30.3%]; median [IQRs] age, 28 [24–38]), with 155 (51.7%) healthy participants and 145 (48.3%) confirmed with community-acquired pneumonia (CAP). Dataset-Nodule (China-Japan Friendship Hospital, China) comprised 752 participants (292 female [38.8%]; median [IQRs] age, 53 [45–63]), including 251 (33.4%) healthy participants and 501 (66.6%) patients with lung nodules (mean [Standard Deviation (SD)] size, 8.7 [3.4] mm). The reference standard of CAP and lung nodule are detailed in Supplementary Note.

**Table 1:** Baseline characteristics of data sets (*N* = 2802)

	No. (%)			
Variable	Dataset- Development (*n* = 1576)	Dataset- USA (*n* = 174)	Dataset- Pneumonia (*n* = 300)	Dataset- Nodule (*n* = 752)
Age, Median (IQRs), y	26 (22–33)	63 (54–71)	28 (24–38)	53 (45–63)
Sex				
M	893 (56.7)	91 (52.3)	209 (69.7)	460 (61.2)
F	683 (43.3)	83 (47.7)	91 (30.3)	292 (38.8)
Normal participants	1576 (100.0)	NA	155 (51.7)	251 (33.4)
CAP patients	NA	NA	145 (48.3)	NA
CAP subtype				
Bacterial	NA	NA	31 (21.4)	NA
Non-Bacterial	NA	NA	114 (78.6)	NA
Nodule patients	NA	NA	NA	501 (66.6)
Total No. of nodules	NA	NA	NA	1567
No. of nodules per patient, Median (Range)	NA	NA	NA	2 (1–57)
Nodule size on CT				
Mean (SD), mm	NA	NA	NA	8.7 (3.4)
3–6 mm	NA	NA	NA	122 (7.8)
6–9 mm	NA	NA	NA	969 (61.8)
9–12 mm	NA	NA	NA	318 (20.3)
12–60 mm	NA	NA	NA	158 (10.1)
Internal characteristics of CT findings				
Solid nodule	NA	NA	NA	968 (61.8)
Subsolid nodule	NA	NA	NA	108 (6.9)
Calcific nodule	NA	NA	NA	491 (31.3)

*IQRs* interquartile ranges, *CAP* community-acquired pneumonia, *NA* not applicable, *CT* computed tomography.

CT scans were acquired using multidetector-row CT scanners from three vendors (UIH, Siemens Healthineers, and Philips, detailed in Supplementary Table 1). Inclusion criteria required participants to have CT with 1-mm and 5-mm slice thicknesses reconstructed from identical raw data. Scans with poor image quality upon manual inspection were excluded (Supplementary Fig. 1).

### Synthetic thin-slice CT generation

**Fig. 1:**
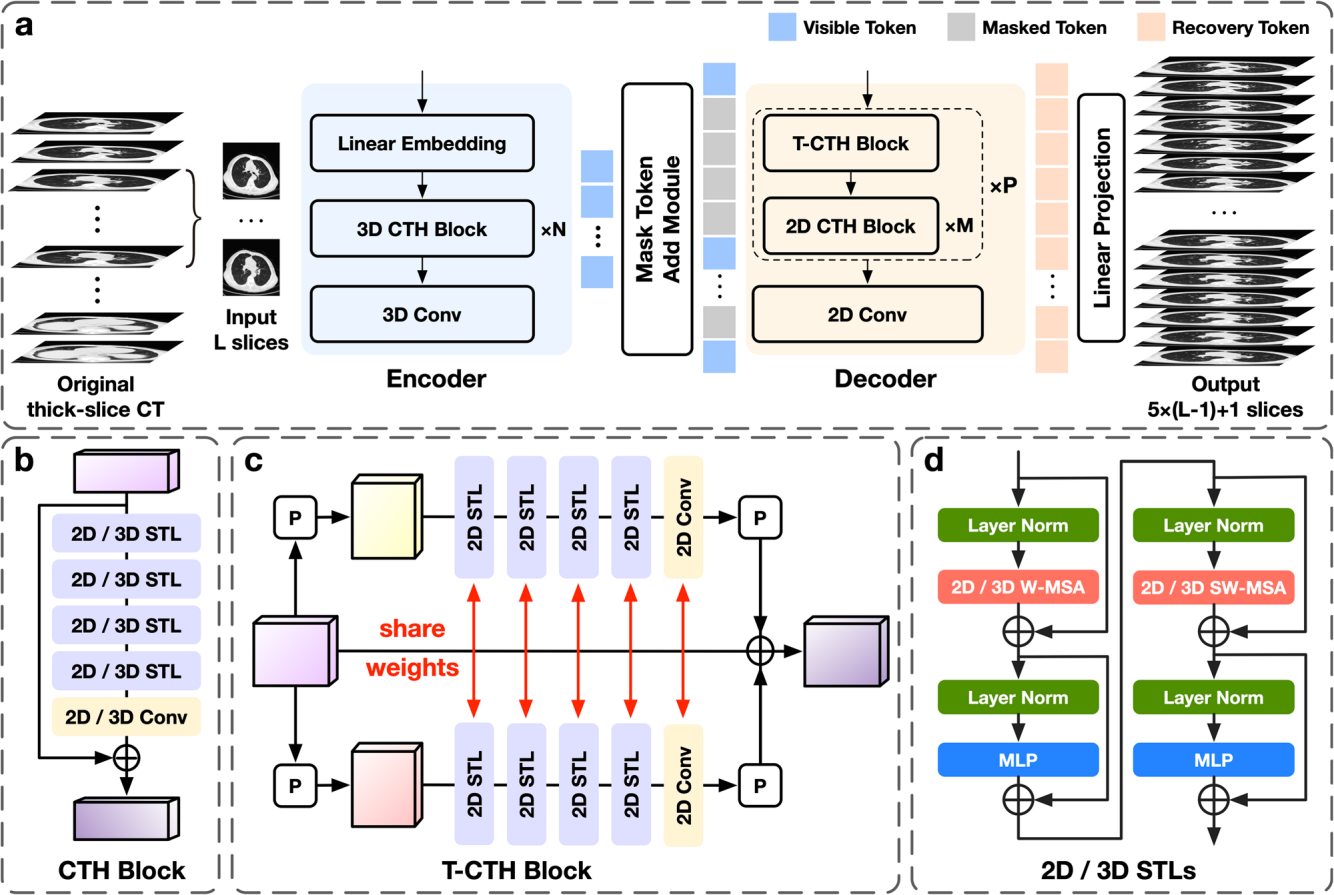
Overview of deep learning synthetic model: a convolutional-transformer hybrid encoder-decoder architecture synthesizes thin-slice CT from thick-slice CT by recovering masked regions from visible regions. **a** The Encoder maps the input L slices from the original thick-slice CT (visible regions) to a latent representation. Masked regions are introduced via the Mask Token Add Module and combined with the latent representation. The Decoder then recovers the masked regions from the latent representation, producing an output size of 5 × (L–1) + 1 through the final Linear Projection. **b** The CTH Block comprises four successive STLs and a Conv. The 3D CTH Block consists of 3D STL and 3D Conv, while the 2D CTH Block consists of 2D STL and 2D Conv. **c** The T-CTH Block has two parallel branches that perform feature extraction from the coronal and sagittal views, respectively. The permutation operation P is used to transform the input view to coronal or sagittal views, or vice versa. **d** Details of two successive 2D or 3D STLs. CTH Block indicates convolutional-transformer hybrid block; T-CTH Block, through-plane convolutional-transformer hybrid block, Conv convolutional, P permutation operation, W-MSA window multi-head self-attention, SW-MSA shift window multi-head self-attention, MLP multi-layer perceptron.

The overview of our DLS model is presented in Fig. 1. During training, cubes of size 8 × 256 × 256 from real 5-mm CT were used as input, and the corresponding cubes of size 36 × 256 × 256 from real 1-mm CT serving as ground truth, where 36 = (8 − 1) × 5 + 1. For inference, we employed a sliding window approach, feeding cubes of size 8×256×256 from the real-5mm CT into the trained DLS model. The axial dimension overlap was set to 1, while overlaps in the other dimensions were set to 0. Multiple predictions for the same coordinate were averaged to obtain the final value. The original thick-slice CT from each test set were processed by the trained DLS model, successfully generating the corresponding synthetic thin-slice CT.

**Fig. 2:**
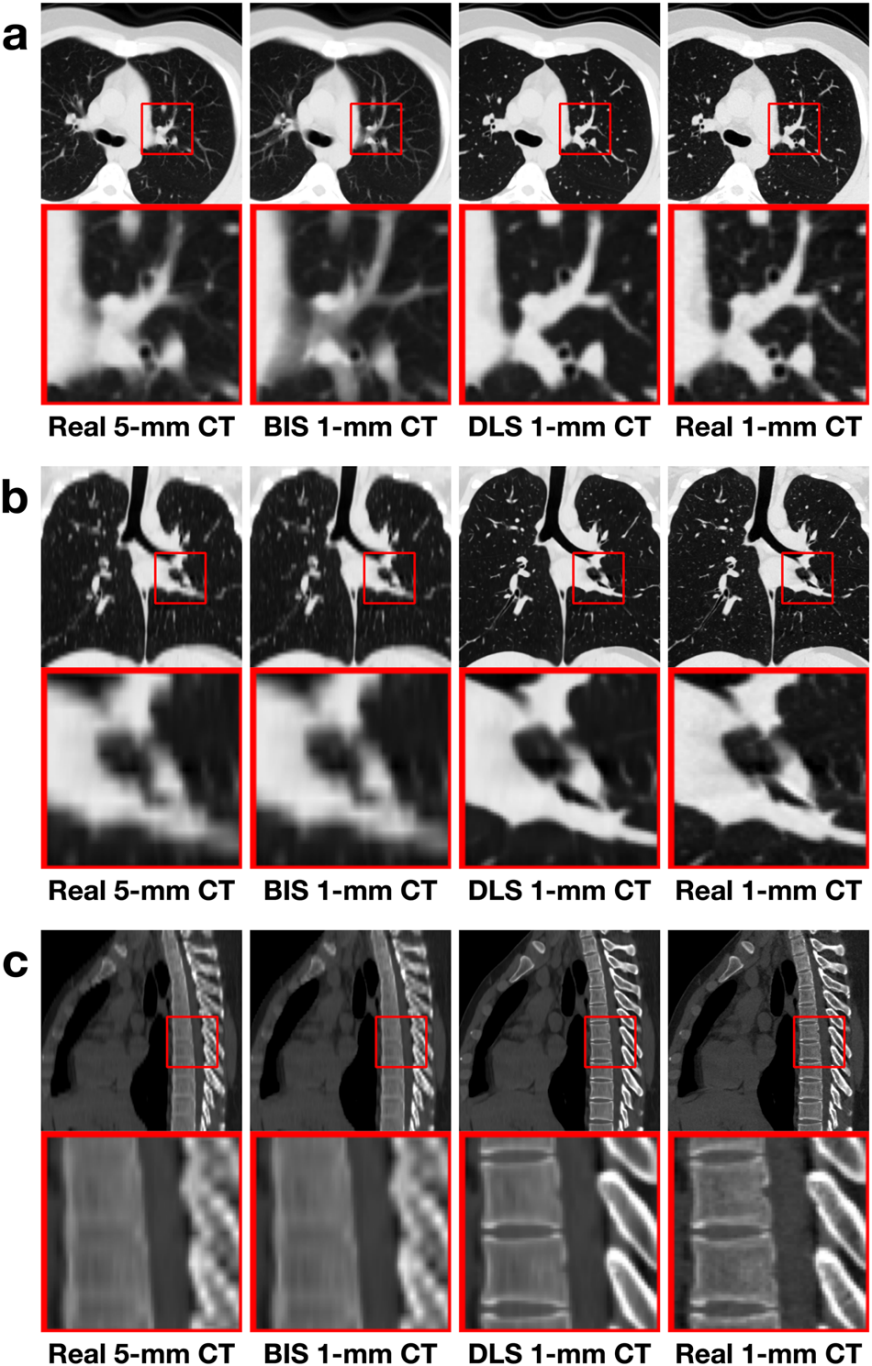
Different CT images for 24-year-old man from dataset-development. **a** Axial view displayed as the lung window. **b** Coronal view displayed as the lung window. **c** Sagittal view displayed as the bone window. BIS indicates bicubic interpolation synthetic; DLS deep learning synthetic.

**Fig. 3:**
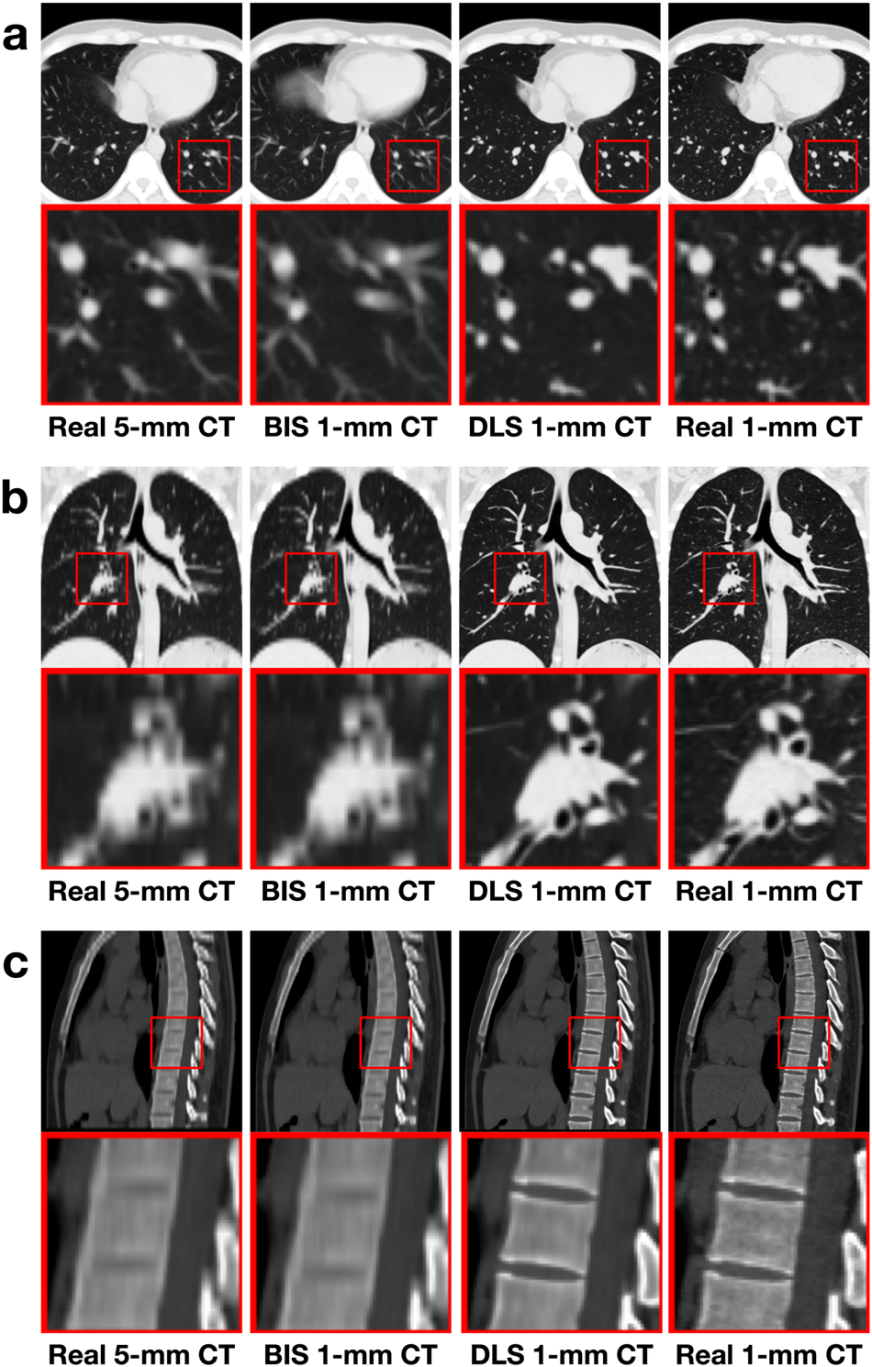
Different CT images for 26-year-old woman from dataset-USA. **a** Axial view displayed as the lung window. **b** Coronal view displayed as the lung window. **c** Sagittal view displayed as the bone window. BIS indicates bicubic interpolation synthetic, DLS deep learning synthetic, CT computed tomography, USA United States of America.

Typical medical image processing tools often employ traditional methods like interpolation resampling to modify image resolution. For comparison, we resampled thick-slice CT in each test set by using SimpleITK (version 2.0, https://simpleitk.org/doxygen/v2_0/html/), resulting in bicubic interpolation-based synthetic (BIS) thin-slice CT^25^. Illustrative examples are shown in Figs. 2, 3 and Supplementary Figs. 2, 3.

### Image quality: quantitative evaluation

**Fig. 4:**
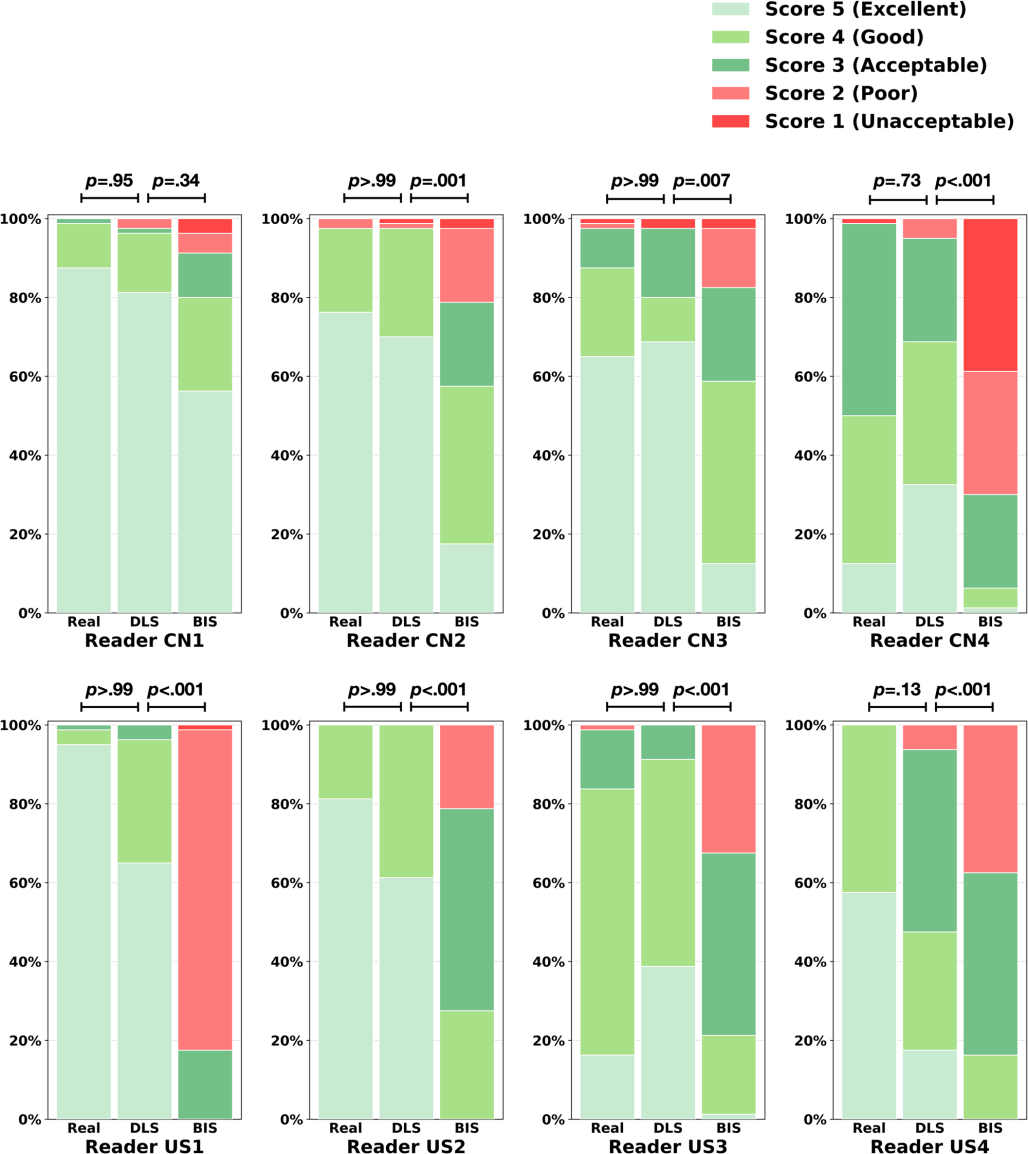
Stacked bar graphs display the distribution of quality scores. Eight radiologists independently rated Real, BIS, and DLS 1-mm CT using a five-point Likert scale (1 = unacceptable, 2 = poor, 3 = acceptable, 4 = good, 5 = excellent). In the Likert scale, scores of ‘unacceptable’ and ‘poor’ are defined as nondiagnostic (displayed in varying shades of red); scores of ‘acceptable’, ‘good’ and ‘excellent’ are defined as diagnostic (displayed in varying shades of green). BIS indicates bicubic interpolation synthetic, DLS deep learning synthetic, CN China, US United States.

Table 2 showed the image quality comparison results in terms of quantitative metrics, including peak signal-to-noise ratio (PSNR)^26^ and structural similarity index measure (SSIM)^27^. DLS 1-mm demonstrated robust performance on internal and external test sets, surpassing traditional BIS 1-mm (all *p* < 0.001). Particularly, DLS 1-mm achieved a median PSNR of 44.08 and SSIM of 0.99 on the internal test set. For external test sets, the PSNRs of Dataset-USA, Dataset-Pneumonia, and Dataset-Nodule were 36.64, 42.95, and 38.69, and SSIMs were 0.92, 0.98, and 0.94, respectively. Compared to several SOTA spatial SR methods, including three CNN-based methods^21–23^ and a Transformer-based method^24^, our DLS model not only had higher PSNR and SSIM in internal and external test sets (all *p* < 0.001), but also demonstrated a better trade-off between quantitative image quality (PSNR and SSIM), running time, and graphics processing unit (GPU) memory (Supplementary Table 2, Fig. 4).

**Table 2:** Quantitative image quality

Variables	BIS 1-mm CT	DLS 1-mm CT	*P* value^a^
Dataset-Development			
PSNR, median [IQRs]	34.31 [33.75–34.91]	44.08 [43.32–44.66]	<0.001
SSIM, median [IQRs]	0.96 [0.95–0.96]	0.99 [0.99–0.99]	<0.001
Dataset-USA			
PSNR, median [IQRs]	31.75 [31.41–32.28]	36.64 [35.65–37.33]	<0.001
SSIM, median [IQRs]	0.87 [0.86–0.89]	0.92 [0.90–0.93]	<0.001
Dataset-Pneumonia			
PSNR, median [IQRs]	34.74 [34.50–35.13]	42.95 [42.46–43.41]	<0.001
SSIM, median [IQRs]	0.95 [0.95–0.96]	0.98 [0.98–0.98]	<0.001
Dataset-Nodule			
PSNR, median [IQRs]	33.73 [33.16–34.37]	38.69 [37.69–39.68]	<0.001
SSIM, median [IQRs]	0.91 [0.89–0.92]	0.94 [0.92–0.95]	<0.001

*IQRs* interquartile ranges, *BIS* bicubic interpolation synthetic, *DLS* deep learning synthetic, *PSNR* peak signal-to-noise ratio, *SSIM* structural similarity index measure, *CT* computed tomography.

^a^*P*-values are derived from the Wilcoxon Signed-Rank test with Bonferroni correction.

For the ablation studies, the results of the first study indicated that our DLS model, trained on 200 samples (20%), outperforms all CNN-based methods trained on all samples (100%). Furthermore, when our DLS model was trained on 500 samples (50%), it demonstrated superior performance compared to the Transformer-based method using all samples (Supplementary Fig. 5). These findings suggest that our DLS model is the most suitable option, even for fine-tuning purposes on new datasets. The results of the second ablation study were shown in Supplementary Table 3. Compared with five vision Transformer-based methods, our DLS model achieves higher PSNR (all *p* < 0.001) and SSIM (all *p* < 0.001).

### Image quality: qualitative evaluation

For qualitative evaluation, 20 participants were randomly chosen from each test set, resulting in 80 participants, to conduct a blinded multi-reader study. CT of three types (Real 1-mm, BIS 1-mm, DLS 1-mm) were included for each participant. Eight radiologists (4–23 years’ experience, four from the USA and four from China) independently rated subjective image quality of each CT scan using a five-point Likert scale (1 indicates unacceptable, 5 indicates excellent, ≥3 indicates diagnostic quality) referring to the European guidelines on quality criteria for CT (https://www.drs.dk/guidelines/ct/quality/htmlindex.htm). Eight radiologists rated Real 1-mm from 3.6 to 4.9, DLS 1-mm from 3.6 to 4.8, and BIS 1-mm from 2.0 to 4.2 (Supplementary Table 4).

For each radiologist, the count of DLS 1-mm rated as the diagnostic quality was non-inferior to Real 1-mm (all *p* > 0.05; Fig. 4). All radiologists’ combined rating was shown in Tables 3, 99.1% (634/640) of real 1-mm, 97.7% (625/640) of DLS 1-mm, and 63.6% (407/640) of BIS 1-mm were rated as the diagnostic quality (Real vs. DLS, *p* = 0.16; Real vs. BIS, *p* < 0.001). For Real 1-mm, most were rated as 5 (excellent, 393 of 640 [61.5%]), followed by 4 (good, 180 of 640 [28.1%]), and the prespecified non-inferiority criterion was 4. DLS 1-mm received ratings of 4 or 5 for 542 of 640 (84.6%) with median [IQRs] score of 5^4,5^, affirming the non-inferiority to Real 1-mm (*p* < 0.001); in contrast, BIS 1-mm did not (median [IQRs], 3^2–4^; *p* > 0.99).

**Table 3:** Image quality assessments according to multi-reader study

Variables	Real 1-mm	DLS 1-mm	BIS 1-mm
Combined five-point (*n* = 640)			
Unacceptable, No. (%)	2 (0.3)	3 (0.5)	39 (6.1)
Poor, No. (%)	4 (0.6)	12 (1.9)	194 (30.3)
Acceptable, No. (%)	61 (9.5)	83 (13.0)	193 (30.2)
Good, No. (%)	180 (28.1)	194 (30.3)	143 (22.3)
Excellent, No. (%)	393 (61.5)	348 (54.3)	71 (11.1)
Combined binary (*n* = 640)			
Nondiagnostic, No. (%)^a^	6 (0.9)	15 (2.3)	233 (36.4)
Diagnostic, No. (%)^b^	634 (99.1)	625 (97.7)	407 (63.6)
Score			
Mean [SD]	4.5 [0.7]	4.4 [0.8]	3.0 [1.1]
Median [IQRs]	5 [4, 5]	5 [4, 5]	3 [2, 4]

*DLS* deep learning synthetic, *BIS* bicubic interpolation synthetic, *CN* China, *US* United States.

^a^Nondiagnostic included unacceptable (score = 1) and poor (score = 2).

^b^Diagnostic included acceptable (score = 3), good (score = 4) and excellent (score = 5).

### Clinical applicability evaluation: CAP diagnostic

The clinical application potential of DLS 1-mm CT was examined through two reader studies of chest diseases, including CAP diagnosis and lung nodule detection. For CAP diagnostic, 100 participants were randomly selected from Dataset-Pneumonia (CAP positive, 50% [50/100]). Four radiologists (3–14 years’ experience) achieved greater accuracy with synthetic thin-slice CT (DLS 1-mm) than original thick-slice CT (Real 5-mm) (Reader 1: 93.0% [93/100] vs. 85.0% [85/100], *p* = 0.02; Reader 2: 89.0% [89/100] vs. 81.0% [81/100], *p* = 0.04; Reader 3: 89.0% [89/100] vs. 79.0% [79/100], *p* = 0.04; Reader 4: 90.0% [90/100] vs. 80.0% [80/100], *p* = 0.004), indicating the utility of synthetic images on CAP diagnosis (Table 4). Three radiologists had higher diagnostic sensitivity using DLS 1-mm than using Real 5-mm (Reader 1: 88.0% [44/50] vs. 76.0% [38/50], *p* = 0.06; Reader 2 80.0% [40/50] vs. 66.0% [33/50], *p* = 0.08; Reader 4: 68.0% [34/50] vs. 80.0% [40/50], *p* = 0.06), while maintaining specificity higher than Real 5-mm (all *p* > 0.05). Reader 3 obtained the same sensitivity but higher specificity using DLS 1-mm compared to using Real 5-mm (92.0% [46/50] vs. 72.0% [36/50], *p* = 0.01). All radiologists also had higher precision and F1-score when using DLS 1-mm than using Real 5-mm (all *p* > 0.05; except for precision of Reader 3, *p* = 0.02). Of note, all radiologists achieved non-inferior accuracy, sensitivity, specificity, precision, and F1-score on DLS 1-mm compared to Real 1-mm (all *p* > 0.99).

**Table 4:** Evaluation of CAP diagnosis

Variables	Real 5-mm	Real 1-mm	DLS 1-mm
Reader 1			
Accuracy, % (No./total No.)	85.0 (85/100)	93.0 (93/100)	93.0 (93/100)
95% CI	78.0–92.0	87.0–97.0	88.0–98.0
*P*-value (vs. DLS 1-mm)	0.02*	>0.99	NA
Sensitivity, % (No./total No.)	76.0 (38/50)	90.0 (45/50)	88.0 (44/50)
95% CI	63.8–87.3	80.4–97.6	78.2–96.0
*P*-value (vs. DLS 1-mm)	0.06	>0.99	NA
Specificity, % (No./total No.)	94.0 (47/50)	96.0 (48/50)	98.0 (49/50)
95% CI	86.0–100.0	90.2–100.0	93.5–100.0
*P*-value (vs. DLS 1-mm)	>0.99	>0.99	NA
Precision, % (No./total No.)	92.7 (38/41)	95.7 (45/47)	97.8 (44/45)
95% CI	82.9–100.0	89.1–100.0	92.7–100.0
*P*-value (vs. DLS 1-mm)	0.50	>0.99	NA
F1-score	83.5	92.8	92.6
95% CI	74.4–91.1	86.0–97.3	86.3–97.4
*P*-value (vs. DLS 1-mm)	0.85	>0.99	NA
Reader 2			
Accuracy, % (No./total No.)	81.0 (81/100)	91.0 (91/100)	89.0 (89/100)
95% CI	73.0–88.0	85.0–96.0	82.0–95.0
*P*-value (vs. DLS 1-mm)	0.04*	>0.99	NA
Sensitivity, % (No./total No.)	66.0 (33/50)	84.0 (42/50)	80.0 (40/50)
95% CI	52.3–78.7	73.1–93.8	68.1–91.1
*P*-value (vs. DLS 1-mm)	0.08	>0.99	NA
Specificity, % (No./total No.)	96.0 (48/50)	98.0 (49/50)	98.0 (49/50)
95% CI	89.6–100.0	93.6–100.0	93.6–100.0
*P*-value (vs. DLS 1-mm)	>0.99	>0.99	NA
Precision, % (No./total No.)	94.3 (33/35)	97.7 (42/43)	97.6 (40/41)
95% CI	84.6–100.0	92.3–100.0	91.9–100.0
*P*-value (vs. DLS 1-mm)	0.70	>0.99	NA
F1-score	77.6	90.3	87.9
95% CI	66.7–86.8	82.9–96.0	79.5–94.4
*P*-value (vs. DLS 1-mm)	0.95	>0.99	NA
Reader 3			
Accuracy, % (No./total No.)	79.0 (79/100)	90.0 (45/50)	89.0 (89/100)
95% CI	70.0–87.0	84.0–95.0	82.0–95.0
*P*-value (vs. DLS 1-mm)	0.04*	>0.99	NA
Sensitivity, % (No./total No.)	86.0 (43/50)	86.0 (43/50)	86.0 (43/50)
95% CI	75.0–95.2	75.6–95.2	75.6–94.8
*P*-value (vs. DLS 1-mm)	>0.99	>0.99	NA
Specificity, % (No./total No.)	72.0 (36/50)	94.0 (47/50)	92.0 (46/50)
95% CI	58.5–84.1	86.4–100.0	83.0–98.2
*P*-value (vs. DLS 1-mm)	0.01	>0.99	NA
Precision, % (No./total No.)	75.4 (43/57)	93.5 (43/46)	91.5 (43/47)
95% CI	63.2–86.2	84.8–100.0	82.2–98.0
*P*-value (vs. DLS 1-mm)	0.02*	>0.99	NA
F1-score	80.4	89.6	88.7
95% CI	71.0–88.0	82.3–95.5	80.9–95.0
*P*-value (vs. DLS 1-mm)	0.78	>0.99	NA
Reader 4			
Accuracy, % (No./total No.)	80.0 (80/100)	89.0 (89/100)	90.0 (90/100)
95% CI	74.0–89.0	83.0–95.0	82.0–95.0
*P*-value (vs. DLS 1-mm)	0.004*	>0.99	NA
Sensitivity, % (No./total No.)	68.0 (35/50)	80.0 (40/50)	80.0 (40/50)
95% CI	53.8–80.9	68.1–91.1	65.3–88.9
*P*-value (vs. DLS 1-mm)	0.06	>0.99	NA
Specificity, % (No./total No.)	91.0 (46/50)	98.0 (49/50)	100.0 (50/50)
95% CI	86.0–100.0	93.5–100.0	100.0–100.0
*P*-value (vs. DLS 1-mm)	0.25	>0.99	NA
Precision, % (No./total No.)	89.5 (34/38)	97.6 (40/41)	100.0 (40/40)
95% CI	78.0–97.6	91.7–100.0	100.0–100.0
*P*-value (vs. DLS 1-mm)	0.22	>0.99	NA
F1-score	77.3	87.9	88.9
95% CI	65.6–86.9	80.0–94.6	81.0–95.1
*P*-value (vs. DLS 1-mm)	0.77	>0.99	NA

*DLS* deep learning synthetic, *CI* confidence interval, *NA* not applicable.

^*^Statistically significant difference at a significance level of *P* < 0.05.

### Clinical applicability evaluation: lung nodule detection

**Fig. 5:**
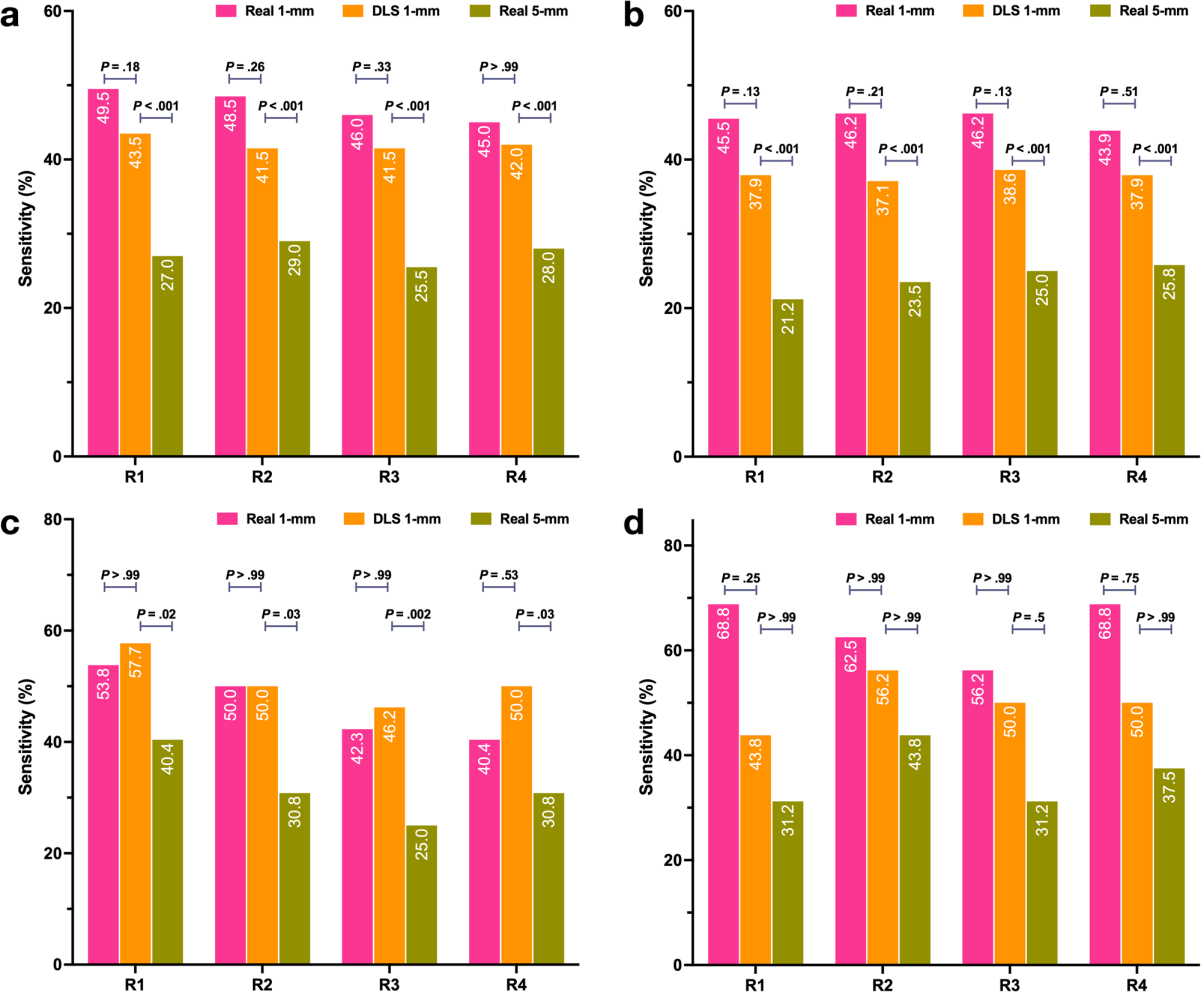
Diagnostic Evaluation of Lung Nodule Detection. Sensitivity of each reader using various types of CT images, shown for **a** all nodules (*N* = 200), **b** solid nodules (*N* = 132), **c** calcific nodules (*N* = 52), and **d** subsolid nodules (*N* = 16). DLS indicates deep learning synthetic, R reader, CT computed tomography.

For lung nodule detection, 84 patients with 200 nodules were randomly selected from Dataset-Nodule, same four radiologists achieved greater nodule detection sensitivity on DLS 1-mm ranging from 41.5% [83/200] to 43.5% [87/200] than that of Real 5-mm which ranged from 25.5% [51/100] to 29.0% [85/100] (all *p* < 0.001; Fig. 5A). For solid nodules, sensitivities on DLS 1-mm (37.1% [49/132] to 38.6% [51/132]) exceeded that on Real 5-mm (21.2% [28/132] to 25.8% [34/132], all *p* < 0.05), and were comparable to that on Real 1-mm (43.9% [58/132] to 46.2% [61/132], all *p* > 0.05), as shown in Fig. 5B. A similar tendency was observed for calcified nodules, sensitivities on DLS 1-mm (46.2% [24/52] to 57.7% [30/52]) surpassed that on Real 5-mm (25.0% [13/52] to 40.4% [21/52], all *p* < 0.05), and was non-inferior to that on Real 1-mm (40.4% [21/52] to 53.8% [28/52], all *p* > 0.05), as shown in Fig. 5C. As for subsolid nodules, Real 1-mm sensitivities (56.2% [9/16] to 68.8% [11/16]) were higher than DLS 1-mm (43.8% [7/16] to 56.2% [9/16]), which in turn outperformed Real 5-mm (31.2% [5/16] to 43.8% [7/16]), albeit not significant (all *p* > 0.05; Fig. 5D).

### Regulated AI-assisted product

**Fig. 6:**
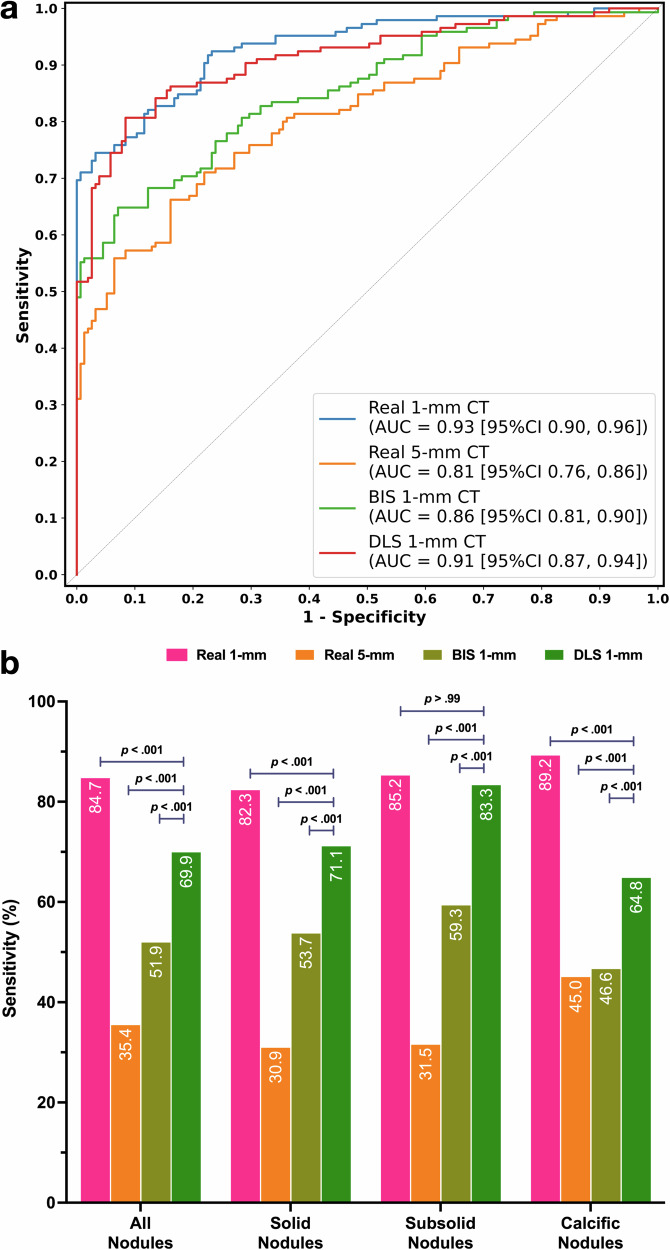
Evaluation of AI-assisted diagnostic product. **a** ROC curves of AI-assisted CAP diagnosis with different CT images. **b** Sensitivity of AI-assisted lung nodule detection with different CT images. AI indicates artificial intelligence, ROC receiver operating characteristic, CAP community-acquired pneumonia, DLS deep learning synthetic, BIS bicubic interpolation synthetic, CT computed tomography.

InferRead CT Pneumonia (Infervision, China), a NMPA-approved AI-assisted product, was employed to evaluated CAP diagnosis performance variability on the Dataset-Pneumonia. We calculated the area under the receiver operating characteristic curve (AUC), sensitivity, specificity, precision, and F1-score using Real 1-mm, Real 5-mm, BIS 1-mm, and DLS 1-mm CT as input, respectively. As shown in Fig. 6A, the AUC of AI-assisted CAP diagnosis varied by CT type: 0.93 with 95% confidence interval (CI) was 0.90 and 0.96 on Real 1-mm, 0.81 (95% CI, 0.76–0.86) on Real 5-mm, and 0.91 (95% CI, 0.87–0.94) on DLS 1-mm. The AUC of DLS 1-mm was superior to Real 5-mm (*p* < 0.001) and non-inferior to Real 1-mm (*p* = 0.42; Table 5). At a specificity of 90.3% [140/155], higher accuracy (85.7% [257/300]), sensitivity (80.7% [117/145]), precision (88.6% [117/132]), and F1-score (84.5) were obtained on DLS 1-mm than Real 5-mm (accuracy, 74.3 [223/300], *p* < 0.001; sensitivity, 57.2 [83/145], *p* < 0.001; precision, 84.7 [83/98], *p* > 0.99; F1-score, 68.3, *p* = 0.02), and comparable to Real 1-mm (all *p* > 0.05).

**Table 5:** CAP diagnosis performance of AI-assisted product

Variable	Value	Pairwise comparisons			
		*P* value	*P* value	*P* value	*P* value
AUC (95% CI)		Real 1-mm	Real 5-mm	BIS 1-mm	DLS 1-mm
Real 1-mm	0.93 (0.90–0.96)	Ref			
Real 5-mm	0.81 (0.76–0.86)	<0.001^*^	Ref		
BIS 1-mm	0.86 (0.81–0.90)	<0.001^*^	0.03^*^	Ref	
DLS 1-mm	0.91 (0.87–0.94)	0.42	<0.001^*^	0.02^*^	Ref
Accuracy, % [No./total No.] (95% CI)		Real 1-mm	Real 5-mm	BIS 1-mm	DLS 1-mm
Real 1-mm	84.0 [252/300] (79.7–88.0)	Ref			
Real 5-mm	74.3 [223/300] (69.3–79.0)	0.001^*^	Ref		
BIS 1-mm	78.0 [234/300] (73.0–82.7)	0.11	0.91	Ref	
DLS 1-mm	85.7 [257/300] (81.3–89.3)	>0.99	<0.001^*^	0.02^*^	Ref
Sensitivity, % [No./total No.] (95% CI)		Real 1-mm	Real 5-mm	BIS 1-mm	DLS 1-mm
Real 1-mm	77.2 [112/145] (70.1–83.8)	Ref			
Real 5-mm	57.2 [83/145] (49.0–64.8)	<0.001^*^	Ref		
BIS 1-mm	64.8 [94/145] (56.8–71.5)	0.013^*^	0.31	Ref	
DLS 1-mm	80.7 [117/145] (73.9–86.8)	>0.99	<0.001^*^	<0.001^*^	Ref
Specificity, % [No./total No.] (95% CI)		Real 1-mm	Real 5-mm	BIS 1-mm	DLS 1-mm
Real 1-mm	90.3 [140/155] (85.4–94.7)	Ref			
Real 5-mm	90.3 [140/155] (85.4–94.7)	>0.99	Ref		
BIS 1-mm	90.3 [140/155] (85.7–94.6)	>0.99	>0.99	Ref	
DLS 1-mm	90.3 [140/155] (85.5–94.7)	>0.99	>0.99	>0.99	Ref
Precision, % [No./total No.] (95% CI)		Real 1-mm	Real 5-mm	BIS 1-mm	DLS 1-mm
Real 1-mm	88.2 [112/127] (82.4–93.3)	Ref			
Real 5-mm	84.7 [83/98] (77.1–91.8)	>0.99	Ref		
BIS 1-mm	86.2 [94/109] (79.8–92.2)	>0.99	>0.99	Ref	
DLS 1-mm	88.6 [117/132] (82.9–93.5)	>0.99	>0.99	>0.99	Ref
F1-score (95% CI)		Real 1-mm	Real 5-mm	BIS 1-mm	DLS 1-mm
Real 1-mm	82.3 (77.4–87.0)	Ref			
Real 5-mm	68.3 (61.3–74.4)	0.04^*^	Ref		
BIS 1-mm	74.0 (67.8–79.4)	0.16	0.28	Ref	
DLS 1-mm	84.5 (79.5–88.8)	>0.99	0.02^*^	0.10	Ref

*CAP* Community-Acquired Pneumonia, *AI* Artificial Intelligence, *AUC* area under the receiver operating characteristic curve, *BIS* bicubic interpolation synthetic, *DLS* deep learning synthetic, *Ref* reference.

^*^Statistically significant difference at a significance level of *P* < 0.05.

InferRead CT Lung (Infervision, China), approved by both FDA and NMPA, evaluated performance variability in lung nodule detection. We calculated the detection sensitivity for various types of nodules in Dataset-Nodule when using Real 1-mm, Real 5-mm, BIS 1-mm, and DLS 1-mm CT as inputs. As shown in Fig. 6B, AI-assisted sensitivity using DLS 1-mm was higher (69.9% [1096/1567]) than Real 5-mm (35.4% [554/1567], *p* < 0.001) but lower than Real 1-mm (84.7% [1327/1567], *p* < 0.001). DLS 1-mm enabled higher sensitivity for solid (71.1% [688/968]) and calcified nodules (64.8% [318/491]) compared to Real 5-mm (solid, 30.9% [299/968], *p* < 0.001; calcific, 45.0% [221/491], *p* < 0.001), yet it was lower than Real 1-mm (solid, 82.3% [797/968], *p* < 0.001; calcific, 89.2% [438/491], *p* < 0.001). For subsolid nodules, DLS 1-mm sensitivity (83.3% [90/108]) exceeded Real 5-mm (31.5% [34/108], *p* < 0.001) and was non-inferior to Real 1-mm (85.2% [92/108], *p* > 0.99; Table 6).

**Table 6:** Lung nodule detection performance of AI-assisted product

Variables	Sensitivity, % [No./total No.]	95% CI, %	Pairwise comparisons			
			*P* value	*P* value	*P* value	*P* value
All Nodules (*N* = 1567)			Real 1-mm	Real 5-mm	BIS 1-mm	DLS 1-mm
Real 1-mm	84.7 [1327/1567]	82.8–86.5	Ref			
Real 5-mm	35.4 [554/1567]	33.0–37.8	<0.001^*^	Ref		
BIS 1-mm	51.9 [813/1567]	49.4–54.4	<0.001^*^	<0.001^*^	Ref	
DLS 1-mm	69.9 [1096/1567]	67.6–72.2	<0.001^*^	<0.001^*^	<0.001^*^	Ref
Solid nodules (*N* = 968)			Real 1-mm	Real 5-mm	BIS 1-mm	DLS 1-mm
Real 1-mm	82.3 [797/968]	80.0–84.8	Ref			
Real 5-mm	30.9 [299/968]	28.1–34.0	<0.001^*^	Ref		
BIS 1-mm	53.7 [520/968]	50.6–56.8	<0.001^*^	<0.001^*^	Ref	
DLS 1-mm	71.1 [688/968]	68.2–73.9	<0.001^*^	<0.001^*^	<0.001^*^	Ref
Subsolid nodules (*N* = 108)			Real 1-mm	Real 5-mm	BIS 1-mm	DLS 1-mm
Real 1-mm	85.2 [92/108]	77.9–91.7	Ref			
Real 5-mm	31.5 [34/108]	23.3–40.7	<0.001^*^	Ref		
BIS 1-mm	59.3 [64/108]	49.5–69.2	<0.001^*^	<0.001^*^	Ref	
DLS 1-mm	83.3 [90/108]	76.4–89.9	>0.99	<0.001^*^	<0.001^*^	Ref
Calcific nodules (*N* = 491)			Real 1-mm	Real 5-mm	BIS 1-mm	DLS 1-mm
Real 1-mm	89.2 [438/491]	86.4–91.7	Ref			
Real 5-mm	45.0 [221/491]	40.5–49.2	<0.001^*^	Ref		
BIS 1-mm	46.6 [229/491]	42.2–51.2	<0.001^*^	>0.99	Ref	
DLS 1-mm	64.8 [318/491]	60.6–69.0	<0.001^*^	<0.001^*^	<0.001^*^	Ref

*AI* Artificial Intelligence, *BIS* bicubic interpolation synthetic, *DLS* deep learning synthetic, *Ref* reference.

^*^Statistically significant difference at a significance level of *p* < 0.05.

## Discussion

Thick-slice CT remain prevalent in clinical practice, particularly in developing countries, where upgrading to advanced CT scanners and expanding data centers is not trivial. The coarse spatial resolution of thick-slice CT challenges radiologists and computer-aided analysis, potentially leading to misdiagnosis or unforeseen consequences. Especially for existing regulated AI-assisted diagnostic products, which often have explicit input constraints, re-developing the product to accommodate differences in slice thickness would incur significant effort and cost. In this study, we developed a DL-based model to generate synthetic thin-slice CT from thick-slice counterparts and accessed the potential for integrating these synthetic images into clinical workflows to overcome the aforementioned disadvantages. To this end, we make the following key contributions: (1) We propose a novel encoder-decoder network with a Convolutional-Transformer hybrid architecture for generating synthetic thin-slice CT from thick-slice CT; (2) We demonstrate that the image quality of synthetic thin-slice CT is comparable to that of real thin-slice CT through multicenter quantitative and qualitative evaluations; (3) We verify the clinical applicability of our method in enhancing the diagnosis of CAP and detection of lung nodules, revealing that radiologist using synthetic thin-slice CT outperform those using original thick-slice CT, and comparable to those using real thin-slice CT; (4) We confirm that synthetic thin-slice CT provides improvements in AI-assisted CAP diagnosis and lung nodule detection compared to the original thick-slice CT.

DL algorithms have proven capable of enhancing medical images across various scenarios^15–20^, including spatial SR to improve image quality of CT. Early methods focused primarily on the quantitative similarity of synthetic thin-slice CT to real thin-slice CT^21–24^. In addition to quantitative image quality, several studies explored potential clinical applications of synthetic thin-slice CT, including radiologist diagnosis^23^ and computer-aided systems^11^. However, integrating synthetic thin-slice CT into clinical practice encounters two obstacles.

The first is the need for large-scale external validation, both quantitative and qualitative. This study externally validated image quality of synthetic thin-slice CT across three centers from two countries. The quantitative assessment showed that our DLS model consistently surpassed other spatial SR methods like bicubic interpolation^25^ and various DL models^21–24^ in terms of PSNR and SSIM. In a qualitative assessment by eight radiologists, no significant differences were observed between synthetic and real thin-slice CT, highlighting the potential of synthetic thin-slice CT as a complementary view to thick-slice CT in diagnosis. The second obstacle is the need to evaluate of synthetic images’ applicability in clinical practice. Liu et al.^23^ demonstrated that radiologists achieve higher sensitivity and precision in lung nodule detection with synthetic thin-slice CT than original thick-slice CT, yet comparisons with real thin-slice CT were absent. Our study compared the real thin-slice CT, synthetic thin-slice CT, and original thick-slice CT through diagnostic evaluation, including CAP diagnosis and lung nodule detection. Results showed that synthetic thin-slice CT significantly outperformed the original thick-slice CT and was comparable to real thin-slice CT. Notably, our DLS model’s training data comprised only healthy participants and were acquired on a CT scanner different from the external test sets, which included patients with complex anomalies. Despite these challenges, the synthetic thin-slice CT maintained high image quality, underscoring the model’s robust generalization and its potential for further improving synthesis quality through diversifying training data or fine-tuning for specific datasets.

Moreover, the potential benefits of synthetic images for AI-assisted diagnostic products deserve attention. Numerous AI-assisted products have received approval from authoritative organizations such as the FDA and NMPA are currently utilized in clinical settings^9,10^.

However, products primarily designed for thin-slice CT often exhibit suboptimal performance with thick-slice CT^11–14^. Consequently, the aforementioned developing countries face disparities in benefiting from AI, exacerbating existing healthcare inequalities. In this study, we first evaluated the performance difference between synthetic CT and real CT using a NMPA-approved AI software for diagnosing CAP. Compared to thick-slice CT, synthetic thin-slice CT exhibited superior performance, which even proved noninferior to real thin-slice CT. Furthermore, we evaluated the performance of lung nodule detection with an FDA-approved AI software, and found that although synthetic thin-slice CT was inferior to real thin-slice CT, it is still much better than thick-slice CT. For subsolid nodules, synthetic thin-slice CT showed noninferior sensitivity compared to real thin-slice CT, which is clinically important as subsolid nodules, particularly part-solid nodules, have a higher malignancy rate than solid nodules^28–30^. Our study revealed a noticeable decrease in the performance of two regulated AI products when using thick-slice CT, whereas synthetic thin-slice CT effectively counteracted this decline, suggesting that our model has definite use and merit in empowering AI-assisted products deployment in regions with scarce medical resources.

This study had limitations. First, this study aimed to externally validate the clinical utility of synthetic thin-slice CT in a multicenter, multiregional setting. However, its generalizability was limited because only the smallest test set came from outside the country of most data sources. Moreover, this preliminary study was restricted to chest CT and did not assess the model’s suitability for other anatomical structures. Future studies will extend the validation across different anatomical structures and diagnostic tasks internationally. Second, our DL model’s evaluation used pairs of thin-slice and thick-slice CTs reconstructed from identical raw data. Therefore, assessing the model’s performance with directly scanned thick-slice CT remains necessary, and this will be incorporated into our continued validation efforts. Third, although our DLS model achieves significantly highest quantitative image quality, its running time lags behind other two DL method^23,24^. This discrepancy may stem from the incorporation of spatial computations in the DLS model to leverage spatial context, consequently elevating the complexity of the model. Reducing running time is pivotal for user experience and facilitate the integration of the synthetic model into clinical workflow, so an important future work is to improve the computational efficiency of our DLS model while maintaining synthetic quality. Lastly, the state-of-the-art diffusion models exhibit superior performance in image synthesis^31^, but their significant computational demand prevented the exploration in this study. For occasional inaccurate synthesis, enhancing robustness through exploring advanced synthesis methods remain a priority for future research.

In conclusion, this multicenter study found that the DLS model generates synthetic thin-slice CT from thick-slice chest CT, yielding images that match the image quality of real thin-slice CT. The synthetic thin-slice CT exhibited good performance in CAP diagnosis and lung nodule detection, superior to the original thick-slice CT and comparable to real thin-slice CT. Furthermore, the performance of AI-assisted diagnosis products that previously underperformed with original thick-slice CT was improved by using synthetic thin-slice CT. With additional research and validation, synthetic thin-slice CT could serve as a practical alternative to real thin-slice CT, especially when the latter is preferable but unavailable. Prospective studies are essential to substantiate these findings.

## Methods

This cross-regional, multicenter study was performed in four centers, including one in the United States and three in China. We developed our DLS model on one center and evaluated synthetic thin-slice CT with internal and external validation on all four centers. Considering the study’s retrospective nature, all participating centers’ institutional review boards (IRB) either approved this study (Beijing Haidian Hospital Medical Ethics Committee, BHHMEC-YJ-2021-02; Ethics Committee of Chinese PLA General Hospital, S2023-498-01; Ethics Committee of China-Japan Friendship Hospital, 2022-KY-127) or exempted it from review (University of California Los Angeles Office of the Human Research Protection Program, IRB#23-001216). When IRB review was performed, written informed consent was waived. All collected CT were deidentified.

### Related work

CNN-based algorithms have demonstrated exceptional performance in SR for natural images^26^, and these techniques have been introduced for spatial SR. Bae et al. were the first to apply CNN to spatial SR, using a 2D-based approach on coronal or sagittal planes and then composing the results into a 3D output^32^. Recognizing the limitations of 2D networks in modeling spatial context, Ge et al. introduced a 3D-based approach to capture expressive volumetric representations with inter-slice correlation, resulting in excellent image quality^21^. Peng et al. proposed a multi-stage 3D method that allows for arbitrary upsampling ratios in spatial SR^22^, later expanding this to a domain-adaptive mode^33^. Chen et al. also explored arbitrary resolution spatial SR using a neural radiance field-based zero-shot framework^34^. Additionally, certain studies have delved into refining spatial SR model via self-supervised learning strategies to mitigate the impact of data quality^35,36^. Despite substantial progress, CNN-based algorithms are still constrained by the inherent limitations of Convolutional operators. One limitation is the potential oversight of content relevance when applying the same Convolutional kernel across diverse regions. In response, Liu et al.^23^ proposed a CNN-based multi-stream architecture that leverages lung segmentation to separately restore different regions, albeit this strategy may not universally apply. Furthermore, the non-local similarity of image content has proven to be a valuable prior in image restoration^37^. However, the local processing nature of Convolutional operators impedes their capacity to effectively model the non-local relationship.

In contrast to CNN-based algorithms, Transformer excel at capturing long-range dependencies and dynamically aggregating feature weights to enhance input-specific feature representations^38^. These capabilities motivated Yu et al. to develop a Transformer-based spatial SR approach, known as the Transformer Volumetric Super-Resolution Network (TVSRN)^24^. TVSRN frames spatial SR as a task of recovering masked regions from visible regions. It adopts an encoder-decoder architecture with Swin-Transformer layer^39^, where the encoder maps the thick-slice CT (visible regions) to a latent representation, and the decoder recovers the thin-slice CT (masked regions) from this latent representation. The Swin-Transformer layer extracts non-local feature through shifted windows, thereby reducing computational complexity to linear in relation to input size, making it more suitable for high-resolution images.

### Deep learning synthetic model

To generate synthetic 1-mm thin-slice CT from real 5-mm thick-slice CT, we developed a DLS model based on an asymmetric encoder-decoder architecture, extending TVSRN with three notable improvements to enhance synthetic CT quality. First, we incorporated a Convolutional layer at the end of each block in the TVSRN, generating a Convolutional-Transformer hybrid module. This design, inspired by Liang et al.^40^, improved model performance and accelerated convergence in our experiments. Second, we eliminated all masking mechanisms from TVSRN, which serve to restrict self-attention computation to within each sub-window during cyclic-shift computation. This mechanism constrains long-range information interaction and introduces extra computation that may impair the model performance for the spatial SR task. Lastly, we replaced the 2D Swin-Transformer layer in TVSRN encoder with a 3D Swin-Transformer layer^41^, facilitating more effective spatial context utilization and resulting in more representative features.

We trained DLS model using the Dataset-Development training set, which consists of paired Real 1-mm and Real 5-mm CT scans from 1000 healthy participants. Prior to input, the intensities of CT scans were normalized from the range [−1024, 2048] to [0, 1]. Data augmentation included random cropping and horizontal flipping. The model was trained with the AdamW optimizer^42^, using an initial learning rate of 0.0003 and a weight decay of 0.0001. The mini-batch size was set to 1 and the max training epoch was 2000. For model checkpoint selection and hyperparameter optimization, we evaluated the PSNR of models on the Dataset-Development validation set (176 healthy participants, from the same center as the training set) every 5 training epochs. The model achieving the highest PSNR on the validation set was selected for evaluation on the test sets. During training, the learning rate was reduced to 1/10 of its current value if three consecutive evaluations showed no improvement, and training was stopped after three reductions in the learning rate. The framework is implemented in PyTorch framework 1.9.0 on an NVIDIA A6000 GPU.

We compared the performance of DLS model with several SOTA spatial SR methods, including three CNN-based methods^21–23^ and TVSRN^24^. Additionally, we conducted two ablation study on the Dataset-Development to deeply analyze our DLS model. The first experiment assessed the effects of employing different amounts of training data (100 [10%], 200 [20%], 500 [50%], 800 [80%], 1000 [100%]). The second experiment compared the performance of our DLS model with various vision Transformer-based image synthetic methods, including IPT^43^, Uformer^44^, Resformer^45^, ART^46^, and ShuffleFormer^47^.

### Image quality evaluation

For quantitative evaluation of synthetic thin-slice CT, we employed two standard objective measures widely used in image generation, i.e. PSNR and SSIM, for our four test sets. The higher the PSNR and SSIM, the better quantitative quality of the synthetic thin-slice CT.

For qualitative evaluation, 20 participants were randomly chosen from each test set to conduct the multi-reader study, resulting in 80 participants. CT scans of three types (real 1-mm, BIS 1-mm, and DLS 1-mm) were included for each participant, resulting in a total of 240 CT scans. To minimize potential biases, the 240 CT scans were randomly ordered and distributed in such a way that no consecutive scans from the same participant were shown to the radiologists. Each radiologist viewed the CT scans in the same order.

### Clinical applicability evaluation

The clinical application potential of DLS 1-mm CT was examined through two reader studies of chest diseases, including CAP diagnosis and lung nodule detection. Four radiologists (3–14 years’ experience, from China) joined in both reader studies and each study was divided into three distinct rounds, with an interval of 1-month as the washout period. For each participant, three types of CT (real 1-mm, real 5-mm, and DLS 1-mm) were anonymized and randomly assigned to one of the three rounds, ensuring that each round featured only one type per participant.

For CAP diagnosis, each radiologist was required to make a diagnosis of CAP based on each CT, blinded to clinical data. The data were selected for this study through stratified random sampling from Dataset-Pneumonia, yielding a balanced set of 100 cases, evenly divided into 50 pneumonia-positive and 50 pneumonia-negative cases.

For lung nodule detection, each radiologist was required to identify all lung nodules on each CT, blinded to clinical data. For this study, 200 nodules were randomly selected from Dataset-Nodule, following this procedure: initially, patients with nodules in Dataset-Nodule were randomized, then sequentially included. During this process, a patient was included if the sum of their nodules and the accumulated total from previously included patients did not exceed 200; otherwise, that patient was bypassed. The enrollment concluded once the total count of nodules from the included patients reached precisely 200. Finally, 84 patients were enrolled.

The clinical application potential of DLS 1-mm CT was further examined by comparing it with real 5-mm and real 1-mm CT on the same two tasks, i.e., CAP diagnosis and lung nodule detection, when used for regulated AI-assisted diagnostic products. InferRead CT Pneumonia (Infervision, China), approved by the NMPA was employed to evaluated CAP diagnosis performance variability. InferRead CT Lung (Infervision, China), approved by both FDA and NMPA, evaluated performance variability in lung nodule detection.

### Evaluation metrics

PSNR is a widely used metric in image processing and computer vision for assessing image or video quality. It quantifies the ratio between the maximum possible power of a signal and the power of the noise corrupting the signal, expressed in decibels. A higher PSNR value signifies superior image quality and reduced distortion. SSIM calculates the structural similarity between the original and processed images by comparing their mean, variance, and covariance of pixel intensities within a local window. SSIM values range from 0 to 1, with a value of 1 signifying identical images and a value of 0 indicating complete dissimilarity.

For qualitative evaluation, radiologists offered a qualitative assessment of diagnostic image quality by scoring on a 5-point Likert-type scale (5 = excellent, 4 = good, 3 = acceptable, 2 = poor, and 1 = unacceptable; ≥3 indicates diagnostic quality) referring to the European guidelines (https://www.drs.dk/guidelines/ct/quality/htmlindex.htm).

The CAP diagnosis performance of radiologists and AI-assisted product were evaluated using accuracy, sensitivity, specificity, precision, F1-score, and AUC (only AI-assisted product). For lung nodule detection, sensitivity was used to evaluate the performance of radiologists and AI-assisted product.

### Statistical analysis

PSNR and SSIM were visualized with box plots and compared using the Wilcoxon Signed-Rank test. For the multi-reader study, the non-inferiority of synthetic to real CT was tested using one-sided Wilcoxon test at a 0.25-point threshold. The count of CT rated diagnostically acceptable was compared using the chi-square test. For CAP diagnosis, the sensitivity and specificity were compared with the McNemar test, the precision and F1-score were compared with the permutation test, and the AUC was compared with the DeLong test. For nodule detection, the sensitivity was calculated and compared with the McNemar test.

Bootstrapping was used to estimate 95% confidence intervals. Pairwise comparisons were conducted with Bonferroni correction by multiplying *p*-values by the number of comparisons. *P* < 0.05 was considered to indicate a statistically significant difference. All analyses were carried out using Statistical Package for Social Sciences (SPSS), version 28.0 (IBM).

## Supplementary information

SUPPLEMENTAL MATERIAL

## Data Availability

The data used for model development of this study are not publicly available by hospital regulations to protect patient privacy. Limited data access is obtainable upon reasonable request by contacting the corresponding author.

## Code availability

The framework is implemented in PyTorch framework 1.9.0 on an NVIDIA A6000 GPU. Code and model weights are available at https://github.com/smilenaxx/CTHNet-for-CT-Slice-Thickness-Reduction.
