# Supplementary material for: Spatial resolution enhancement using deep learning improves chest disease diagnosis based on thick slice CT
Source: NPJ Digit Med. 2024 Nov 23;7:335. doi: 10.1038/s41746-024-01338-8 (PMC11585608; doi:10.1038/s41746-024-01338-8)
Supplement: Supplementary file 1 — SUPPLEMENTAL MATERIAL [file 41746_2024_1338_MOESM1_ESM.pdf]

Supplementary Note: Reference standard.

Supplementary Figure 1: Flow diagrams for data sets.

Supplementary Figure 2: Different CT images for 26-year-old man from Dataset-Pneumonia.

Supplementary Figure 3: Different CT images for 42-year-old man from Dataset-Nodule.

Supplementary Figure 4: Performance Comparison Scatter Plot.

Supplementary Figure 5: Ablation study of deep learning models.

Supplementary Table 1: Detailed CT imaging protocol information.

Supplementary Table 2: Performance comparison of Spatial SR methods.

Supplementary Table 3: Performance comparison of Transformer methods.

Supplementary Table 4: Image quality assessment of each reader.

Supplementary Checklist: STARD Checklist

Supplementary Note: Reference standard

Annotation for community-acquired pneumonia

For Dataset-Pneumonia, community-acquired pneumonia (CAP) patients and healthy participants were identified through clinical evaluations, laboratory tests, and imaging examinations. The CT image features of patients within the Dataset-Pneumonia included such as consolidation, clustered patchy opacities, nodular shadow, and more. All CAP patients received laboratory confirmation: 21.3% (31/145) were confirmed to be bacterial culture positive, and 78.7% (114/145) were confirmed to be negative (all viral pneumonia).

According to the predefined diagnostic sensitivity of 85% and specificity of 90%, Type I error is 0.05, Error Tolerance (L) is 0.1, and the case-control ratio is 1:1. The formula for calculating the sample size is:

$$Sample\ size\ (n) = \frac{Z_{1-\alpha/2}^{2} \times S \times (1-S)}{L^{2} \times Prevalence}$$

Based on the predefined sensitivity, a minimum sample size of 98 was calculated. Based on the predefined specificity, a minimum sample size of 69 was calculated. Therefore, it is determined that the final minimum sample size of the diagnostic study is 98.

Annotation for lung nodules

For the lung nodule detection analysis, 501 of 752 participants (66.6%, with 1567 nodules) were annotated in previous work, and other healthy participants were excluded. Radiologist M.L. (20 years of chest imaging experience, author) annotated the ground truth that fully enclosed the nodules' region on each slice and classified the lung nodules into three categories: solid, subsolid, and calcified [1].

For DLS 1-mm and BIS 1-mm CT, the lung nodule annotations from Real 1-mm CT could be reused directly. For Real 5-mm CT, the lung nodule annotations of Real 1-mm CT were applied according to the alignment relationship of spatial positions.

Reference

[1] Ettinger DS, Wood DE, Aggarwal C, et al. NCCN Guidelines Insights: Non-Small Cell Lung Cancer, Version 1.2020. J Natl Compr Canc Netw 2019;17(12):1464–1472.

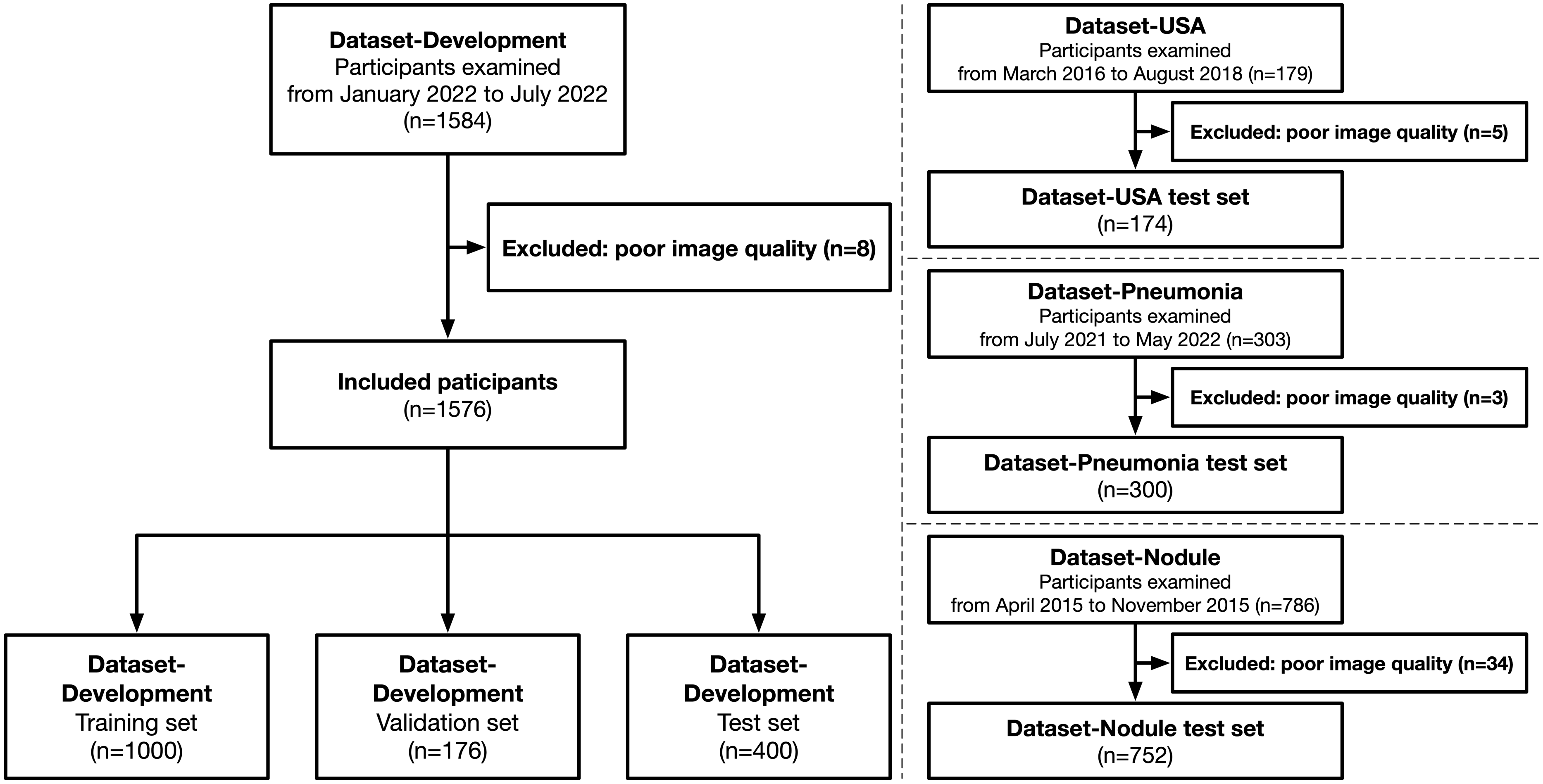

Supplementary Figure 1: Flow diagrams for data sets. USA indicates United States of America.

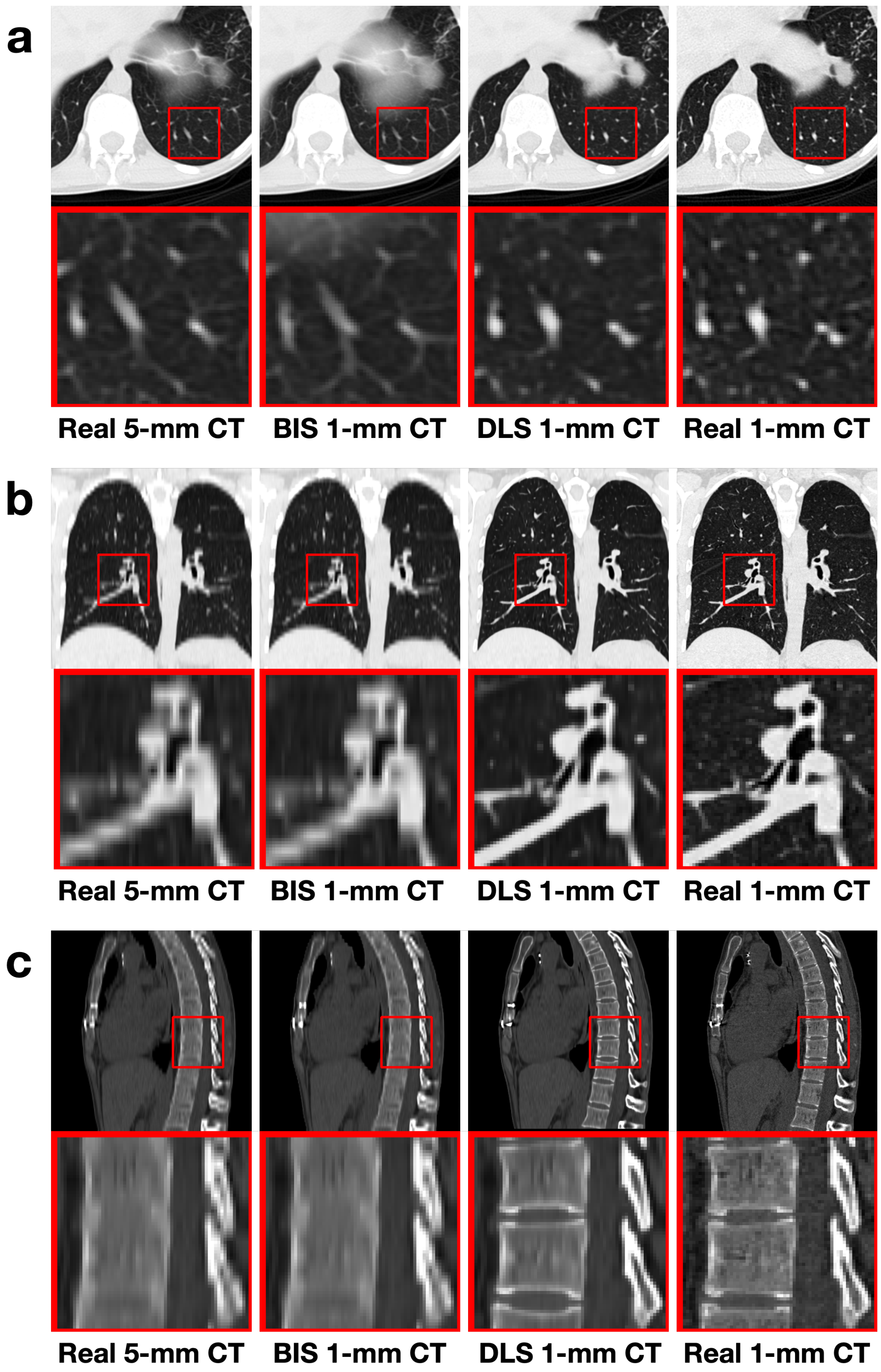

Supplementary Figure 2: Different CT images for 26-year-old man from Dataset-Pneumonia. (a) Axial view displayed as the lung window. **(b)** Coronal view displayed as the lung window. **(c)** Sagittal view displayed as the bone window. BIS indicates bicubic interpolation synthetic; DLS, deep learning synthetic; CT, computed tomography.

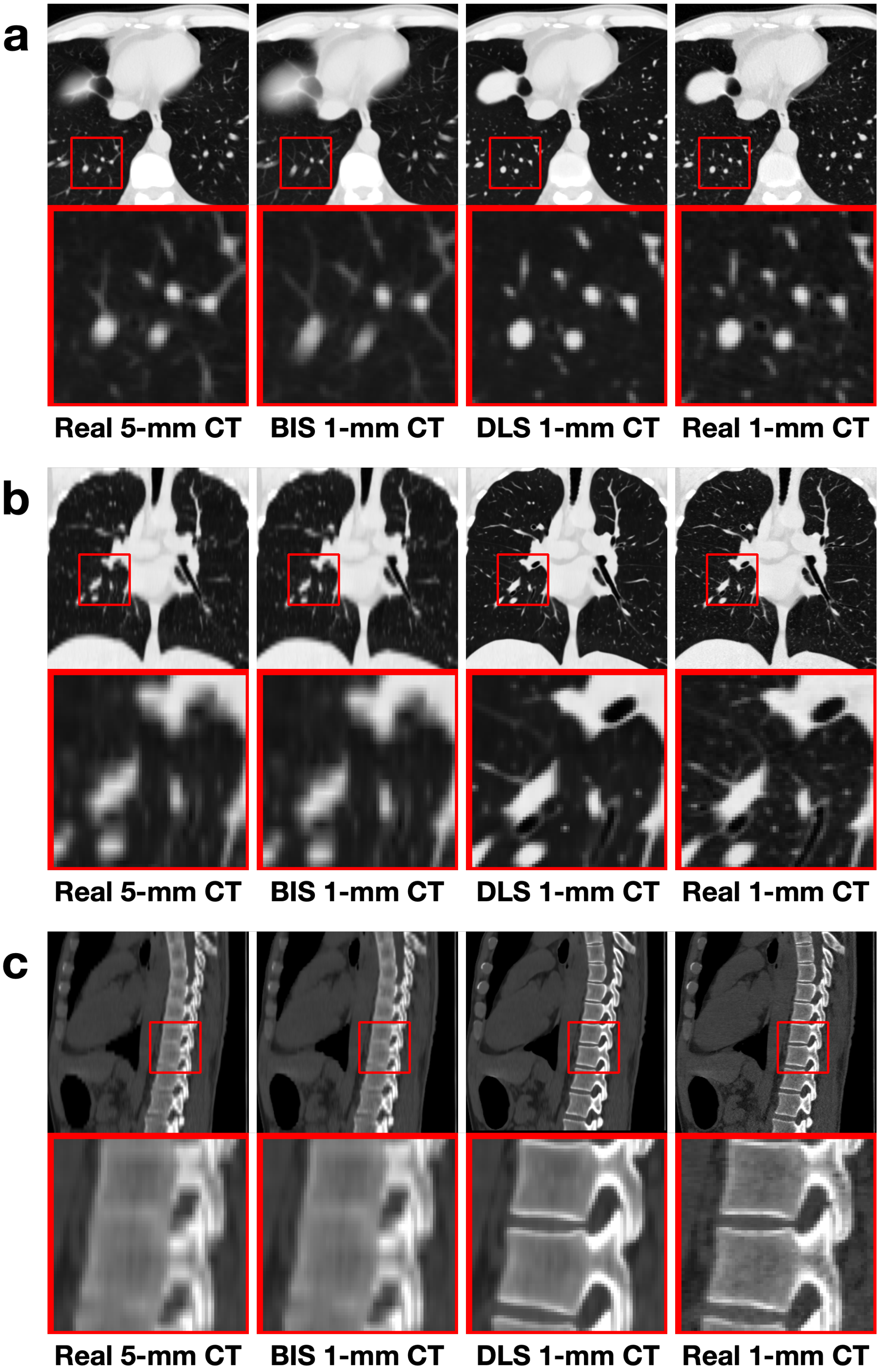

Supplementary Figure 3: Different CT images for 42-year-old man from Dataset-Nodule. **(a)** Axial view displayed as the lung window. **(b)** Coronal view displayed as the lung window. **(c)** Sagittal view displayed as the bone window. BIS indicates bicubic interpolation synthetic; DLS, deep learning synthetic; CT, computed tomography.

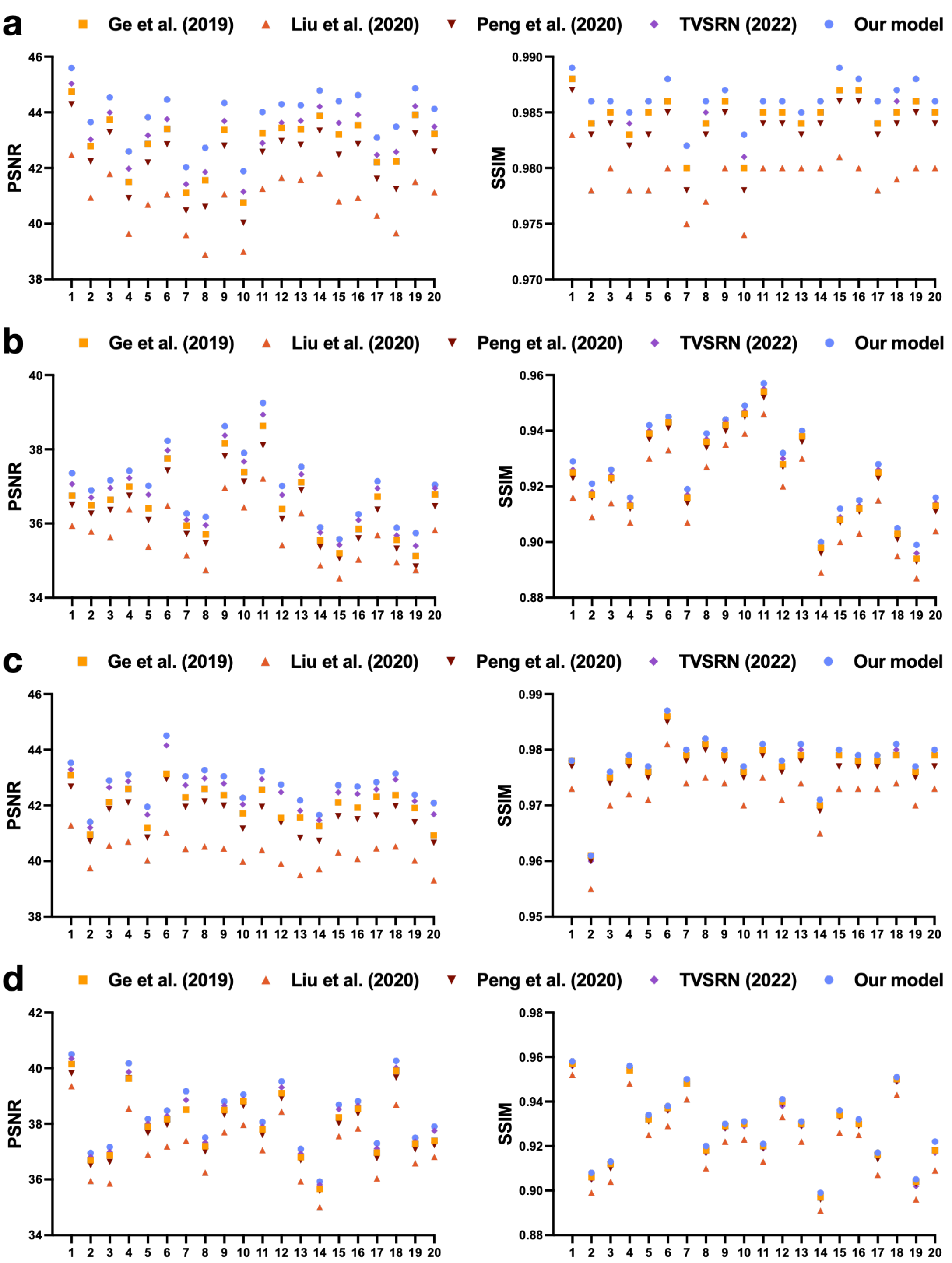

Supplementary Figure 4: Performance Comparison Scatter Plot. For test set **(a)** Dataset-Development, **(b)** Dataset-USA, **(c)** Dataset-Pneumonia, and **(d)** Dataset-Nodule, 20 samples were randomly selected, and the PSNR and SSIM of each sample, corresponding to various deep learning models, are presented as scatter plots. PSNR indicates peak signal-to-noise ratio; SSIM, structural similarity index measure; TVSRN, transformer volumetric super-resolution network.

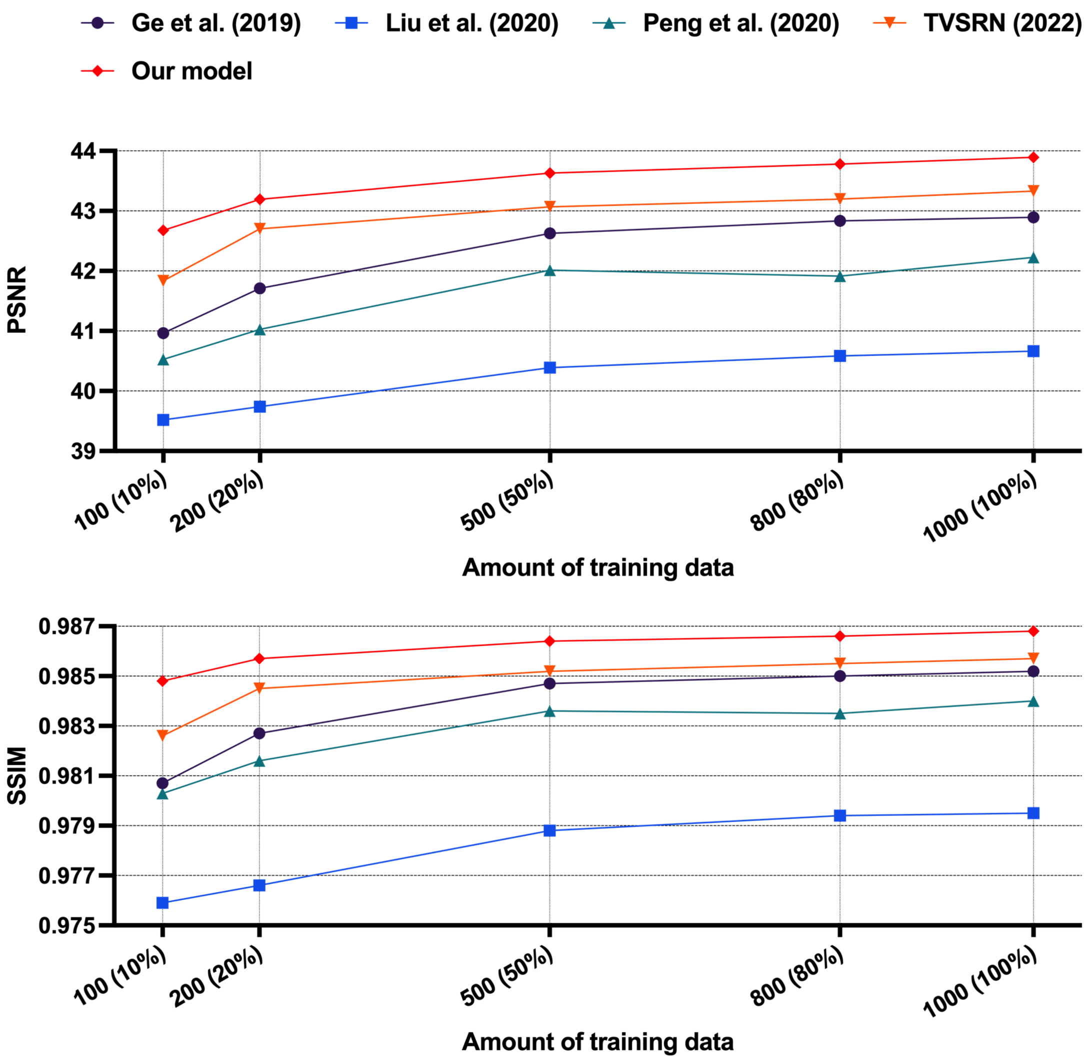

Supplementary Figure 5: Ablation study of deep learning models. PSNR indicates peak signal-to-noise ratio; SSIM, structural similarity index measure.

Supplementary Table 1: Detailed CT imaging protocol information

Dataset	Manufacturer and Scanner	Number of Scans	KVP	Exposure	In-plane pixel resolution (mm^2)
Dataset-Development	UIH uCT 530	1576	80 - 120	13 - 209	0.45 × 0.45 – 0.96 × 0.96
Dataset-USA	Siemens Definition AS	174	120	68 - 398	0.56 × 0.56 – 0.88 × 0.88
Dataset-Pneumonia	Philips iCT 256	300	100 - 120	55 - 404	0.48 × 0.48 – 0.98 × 0.98
Dataset-Nodule	Philips iCT 256	479	120	50 - 300	0.60 × 0.60 – 0.80 × 0.80
Dataset-Nodule	Philips Ingenuity CT	273	120	50 - 200	0.61 × 0.61 – 0.78 × 0.78

Supplementary Table 2: Performance comparison of spatial SR methods

**Variables**	**Ge et al. (2019)**	**Liu et al. (2020)**	**Peng et al. (2020)**	**TVSRN (2022)**	**Our model**
**Dataset-Development**					
PSNR, Mean [SD]	42.895 [1.063] *	40.664 [0.962] *	42.225 [1.103] *	43.199 [1.074] *	43.895 [1.045]
SSIM, Mean [SD]	0.985 [0.002] *	0.980 [0.002] *	0.984 [0.002] *	0.985 [0.002] *	0.987 [0.002]
**Dataset-USA**					
PSNR, Mean [SD]	36.029 [1.238] *	35.259 [0.974] *	35.779 [1.172] *	36.302 [1.242] *	36.504 [1.275]
SSIM, Mean [SD]	0.911 [0.028] *	0.902 [0.028] *	0.909 [0.028] *	0.911 [0.028] *	0.914 [0.028]
**Dataset-Pneumonia**					
PSNR, Mean [SD]	42.166 [0.828] *	40.386 [0.609] *	41.750 [0.777] *	42.591 [0.814] *	42.880 [0.827]
SSIM, Mean [SD]	0.978 [0.006] *	0.973 [0.006] *	0.977 [0.006] *	0.978 [0.006] *	0.979 [0.006]
**Dataset-Nodule**					
PSNR, Mean [SD]	38.402 [1.574] *	37.526 [1.387] *	38.213 [1.498] *	38.609 [1.585] *	38.786 [1.626]
SSIM, Mean [SD]	0.933 [0.022] *	0.925 [0.023] *	0.932 [0.022] *	0.932 [0.023] *	0.934 [0.022]
**Running Time, Mean**	21.4	4.1	50.6	13.9	20.1
**GPU Memory (GB)**	19.4	3.9	8.4	4.2	5.0
**Trainable Parameters (MB)**	3.4	11.8	3.1	10.5	10.6

SR super-resolution, PSNR peak signal-to-noise ratio, SSIM structural similarity index measure, SD standard deviation, GPU graphics processing unit, GB Gigabits, MB Megabits.

* Statistically significant (Wilcoxon Signed-Rank Test with Bonferroni correction, $p < 0.001$) against our model.

Supplementary Table 3: Performance comparison of Transformer methods

**Methods**	**PSNR, Mean [SD]**	**SSIM, Mean [SD]**	**Running Time, Mean**	**GPU Memory (GB)**	**Trainable Parameters (MB)**
IPT	43.054 [1.083] *	0.985 [0.002] *	34.9	8.1	17.0
Uformer	43.264 [1.094] *	0.986 [0.002] *	17.3	4.9	89.8
Resformer	43.227 [1.095] *	0.986 [0.002] *	19.9	4.3	54.0
ART	43.619 [1.056] *	0.986 [0.002] *	20.7	6.3	8.2
ShuffleFormer	43.579 [1.058] *	0.986 [0.002] *	27.0	4.9	89.9
DLS model (ours)	43.895 [1.045]	0.987 [0.002]	20.1	5.0	10.59

DLS deep learning synthetic, PSNR peak signal-to-noise ratio, SSIM structural similarity index measure, SD standard deviation, GPU graphics processing unit, GB Gigabits, MB Megabits.

* Statistically significant (Wilcoxon Signed-Rank test, $p < 0.001$) against our DLS model.

Supplementary Table 4: Image quality assessment of each reader

Variables	Real 1-mm	DLS 1-mm	BIS 1-mm
**CN Reader 1**			
**Score, Mean [SD]**	4.9 [0.4]	4.8 [0.6]	4.2 [1.1]
**Binary (n = 80)**			
Nondiagnostic, No. (%) [a]	0 (0)	2 (3)	7 (9)
Diagnostic, No. (%) [b]	80 (100)	78 (97)	73 (91)
**Five-Point (n = 80)**			
Unacceptable, No. (%)	0 (0)	0 (0)	3 (4)
Poor, No. (%)	0 (0)	2 (3)	4 (5)
Acceptable, No. (%)	1 (1)	1 (1)	9 (11)
Good, No. (%)	9 (11)	12 (15)	19 (24)
Excellent, No. (%)	70 (88)	65 (81)	45 (56)
**CN Reader 2**			
**Score, Mean [SD]**	4.7 [0.6]	4.6 [0.7]	3.5 [1.1]
**Binary (n = 80)**			
Nondiagnostic, No. (%) [a]	2 (3)	2 (3)	17 (21)
Diagnostic, No. (%) [b]	78 (97)	78 (97)	63 (79)
**Five-Point (n = 80)**			
Unacceptable, No. (%)	0 (0)	1 (1)	2 (3)
Poor, No. (%)	2 (3)	1 (1)	15 (19)
Acceptable, No. (%)	0 (0)	0 (0)	17 (21)
Good, No. (%)	17 (21)	22 (28)	32 (40)
Excellent, No. (%)	61 (76)	56 (70)	14 (17)
**CN Reader 3**			
**Score, Mean [SD]**	4.5 [0.8]	4.4 [0.9]	3.5 [1.0]
**Binary (n = 80)**			
Nondiagnostic, No. (%) [a]	2 (3)	2 (3)	14 (18)
Diagnostic, No. (%) [b]	78 (97)	78 (97)	66 (82)
**Five-Point (n = 80)**			
Unacceptable, No. (%)	1 (1)	2 (3)	2 (3)
Poor, No. (%)	1 (1)	0 (0)	12 (15)
Acceptable, No. (%)	8 (10)	14 (17)	19 (24)
Good, No. (%)	18 (23)	9 (11)	37 (46)
Excellent, No. (%)	52 (65)	55 (69)	10 (12)
**CN Reader 4**			
**Score, Mean [SD]**	3.6 [0.8]	4.0 [0.9]	2.0 [1.0]
**Binary (n = 80)**			
Nondiagnostic, No. (%) [a]	1 (1)	4 (5)	56 (70)
Diagnostic, No. (%) [b]	79 (99)	76 (95)	24 (30)
**Five-Point (n = 80)**			
Unacceptable, No. (%)	1 (1)	0 (0)	31 (39)
Poor, No. (%)	0 (0)	4 (5)	25 (31)
Acceptable, No. (%)	39 (49)	21 (26)	19 (24)
Good, No. (%)	30 (38)	29 (36)	4 (5)
Excellent, No. (%)	10 (12)	26 (33)	1 (1)

Supplementary Table 4: Image quality assessment of each reader (continue)

**US Reader 1**			
**Score, Mean [SD]**	4.9 [0.3]	4.6 [0.6]	2.2 [0.4]
**Binary (n = 80)**			
Nondiagnostic, No. (%) [a]	0 (0)	0 (0)	66 (83)
Diagnostic, No. (%) [b]	80 (100)	80 (100)	14 (17)
**Five-Point (n = 80)**			
Unacceptable, No. (%)	0 (0)	0 (0)	1 (1)
Poor, No. (%)	0 (0)	0 (0)	65 (81)
Acceptable, No. (%)	1 (1)	3 (4)	14 (17)
Good, No. (%)	3 (4)	25 (31)	0 (0)
Excellent, No. (%)	76 (95)	52 (65)	0 (0)
**US Reader 2**			
**Score, Mean [SD]**	4.8 [0.4]	4.6 [0.5]	3.1 [0.7]
**Binary (n = 80)**			
Nondiagnostic, No. (%) [a]	0 (0)	0 (0)	17 (21)
Diagnostic, No. (%) [b]	80 (100)	80 (100)	63 (79)
**Five-Point (n = 80)**			
Unacceptable, No. (%)	0 (0)	0 (0)	0 (0)
Poor, No. (%)	0 (0)	0 (0)	17 (21)
Acceptable, No. (%)	0 (0)	0 (0)	41 (51)
Good, No. (%)	15 (19)	31 (39)	22 (28)
Excellent, No. (%)	65 (81)	49 (61)	0 (0)
**US Reader 3**			
**Score, Mean [SD]**	4.0 [0.6]	4.3 [0.6]	2.9 [0.8]
**Binary (n = 80)**			
Nondiagnostic, No. (%) [a]	1 (1)	0 (0)	26 (33)
Diagnostic, No. (%) [b]	79 (99)	80 (100)	54 (67)
**Five-Point (n = 80)**			
Unacceptable, No. (%)	0 (0)	0 (0)	0 (0)
Poor, No. (%)	1 (1)	0 (0)	26 (33)
Acceptable, No. (%)	12 (15)	7 (9)	37 (46)
Good, No. (%)	54 (68)	42 (53)	16 (20)
Excellent, No. (%)	13 (16)	31 (39)	1 (1)
**US Reader 4**			
**Score, Mean [SD]**	4.6 [0.5]	3.6 [0.8]	2.8 [0.7]
**Binary (n = 80)**			
Nondiagnostic, No. (%) [a]	0 (0)	5 (6)	30 (38)
Diagnostic, No. (%) [b]	80 (100)	75 (94)	50 (62)
**Five-Point (n = 80)**			
Unacceptable, No. (%)	0 (0)	0 (0)	0 (0)
Poor, No. (%)	0 (0)	5 (6)	30 (38)
Acceptable, No. (%)	0 (0)	37 (46)	37 (46)
Good, No. (%)	34 (42)	24 (30)	13 (16)
Excellent, No. (%)	46 (56)	14 (18)	0 (0)

DLS deep learning synthetic, BIS bicubic interpolation synthetic, CN China, US United States, SD standard deviation.

[a] Nondiagnostic included unacceptable (score = 1) and poor (score = 2).

[b] Diagnostic included acceptable (score = 3), good (score = 4) and excellent (score = 5).

Supplementary Checklist

STARD 2015 Checklist

Section & Topic	No.	Item	✔ (N/A)
TITLE OR ABSTRACT			
	1	Identification as a study of diagnostic accuracy using at least one measure of accuracy (such as sensitivity, specificity, predictive values, or AUC)	✔
ABSTRACT			
	2	Structured summary of study design, methods, results, and conclusions (for specific guidance, see STARD for Abstracts)	✔
INTRODUCTION			
	3	Scientific & clinical background, including the intended use and clinical role of the index test	✔
	4	Study objectives and hypotheses	✔
METHODS			
Study design	5	Whether data collection was planned before the index test and reference standard were performed (prospective study) or after (retrospective study)	✔
Participants	6	Eligibility criteria	✔
	7	On what basis potentially eligible participants were identified (such as symptoms, results from previous tests, inclusion in registry)	✔
	8	Where and when potentially eligible participants were identified (setting, location and dates)	✔
	9	Whether participants formed a consecutive, random or convenience series	✔
Test methods	10a	Index test, in sufficient detail to allow replication	✔
	10b	Reference standard, in sufficient detail to allow replication	✔
	11	Rationale for choosing the reference standard (if alternatives exist)	✔
	12a	Definition of and rationale for test positivity cut-offs or result categories of the index test, distinguishing pre-specified from exploratory	✔
	12b	Definition of and rationale for test positivity cut-offs or result categories of the reference standard, distinguishing pre-specified from exploratory	✔
	13a	Whether clinical information and reference standard results were available to the performers/readers of the index test	✔
	13b	Whether clinical information and index test results were available to the assessors of the reference standard	✔
Analysis	14	Methods for estimating or comparing measures of diagnostic accuracy	✔
	15	How indeterminate index test or reference standard results were handled	✔
	16	How missing data on the index test and reference standard were handled	✔
	17	Any analyses of variability in diagnostic accuracy,	✔

		distinguishing pre-specified from exploratory	
	18	Intended sample size and how it was determined	✔
**RESULTS**			
Participants	19	Flow of participants, using a diagram. Include the figure number (preferably figure 1) or page number	✔
	20	Baseline demographic and clinical characteristics of participants	✔
	21a	Distribution of severity of disease in those with the target condition	✔
	21b	Distribution of alternative diagnoses in those without the target condition	✔
	22	Time interval and any clinical interventions between index test and reference standard	N/A
Test results	23	Cross tabulation of the index test results (or their distribution) by the results of the reference standard	✔
	24	Estimates of diagnostic accuracy and their precision (such as 95% confidence intervals)	✔
	25	Any adverse events from performing the index test or the reference standard	N/A
**DISCUSSION**			
	26	Study limitations, including sources of potential bias, statistical uncertainty, and generalisability	✔
	27	Implications for practice, including the intended use and clinical role of the index test	✔
**OTHER INFORMATION**			
	28	Registration number and name of registry	N/A
	29	Where the full study protocol can be accessed	N/A
	30	Sources of funding and other support; role of funders	N/A

1
